# Supervised and covariate-adjusted dimension reduction for prediction and visualization using microbiome data

**DOI:** 10.1093/bib/bbag590

**Published:** 2026-10-27

**Authors:** Crystal Zang, Lu Tang, Rebecca A Deek

**Affiliations:** Department of Biostatistics and Health Data Science, University of Pittsburgh, 130 De Soto Street, 15261 PA, United States; Department of Biostatistics and Health Data Science, University of Pittsburgh, 130 De Soto Street, 15261 PA, United States; Department of Biostatistics and Health Data Science, University of Pittsburgh, 130 De Soto Street, 15261 PA, United States

**Keywords:** metagenomics, microbiome, principal coordinates analysis (PCoA), supervised dimension reduction, prediction, visualization

## Abstract

Given that the gut microbiome has been shown to play a critical role in human health, discerning the relationship between microbial taxa and disease outcomes or phenotypes is of interest. Often, this involves association testing, predictive modeling, and visualizing trends in the data. Principal coordinates analysis (PCoA) is a commonly used dimension reduction technique in microbiome studies. PCoA relies on pairwise distances between samples and serves as a nonlinear alternative to the classic principal component analysis when non-Euclidean distances are used. Its low-dimensional embeddings provide intuitive visualizations but do not incorporate outcome-related information. Therefore, the low-dimensional principal coordinates may have poor performance in visualizing outcome differences, as well as in downstream disease prediction tasks. Additionally, covariates, such as demographics, lifestyle factors, and technical artifacts, can obscure true biological signals, leading to masked or spurious associations and limited generalizability. To address these limitations, we propose a supervised and covariate adjusted PCoA algorithm. By utilizing outcome information, while simultaneously minimizing the influence of nuisance variables, our method produces low-dimensional embeddings that better separate disease outcomes in visualizations and result in improved accuracy in downstream regression and classification tasks. We demonstrate the effectiveness of the proposed method and highlight its ability to extract important associations between the microbiome and disease outcomes while mitigating nuisance variation through simulations and application to real microbiome profiling studies.

## Introduction

The human microbiota refers to the trillions of microorganisms, including bacteria, fungi, viruses, and archaea, that inhabit various sites of the body, such as the gut, skin, oral cavity, and respiratory tract. These microbial communities play an essential role in digestion, vitamin production, immune system development, and protection against pathogens [1, 2]. In particular, the gut microbiota has been shown to influence host metabolism [3, 4], inflammation [5], and even brain function through the gut–brain axis [6]. Recent advances have revealed significant associations and potential mechanistic insights regarding a vast array of complex, chronic diseases, including cancer [7, 8], autoimmune disorders [9, 10], and metabolic syndrome [11, 12].

The first step in many epidemiological microbiome studies is to visualize and understand patterns in the complex, high-dimensional microbial features. Given that a single sample can generate information on hundreds to thousands of microbes, dimension reduction techniques are often necessary. Principal coordinates analysis (PCoA), or classical multidimensional scaling, is a generalization of principal component analysis (PCA) that can accommodate non-Euclidean distances [13, 14]. PCoA has been widely applied in biological and ecological studies [15, 16], including the analysis of microbial sequencing data [17]. For microbial profiling data, dissimilarity metrics, such as Bray–Curtis, UniFrac, Jaccard, and Aitchison, are often used to measure pairwise distances, known as *β *-diversity, between samples. PCoA has played a critical role in uncovering patterns in the microbiome’s composition, including identifying biologically meaningful clusters or “enterotypes” [1, 18]. PCoA biplots are also often used as a companion visualization for global tests of *β *-diversity, such as PERMANOVA or MiRKAT [19, 20]. However, PCoA is inherently unsupervised and does not incorporate external information on outcomes or demographic and technical factors into the embedding process. Accordingly, it may identify dimensions that are strongly associated with nuisance variables (e.g. diet or batch) but not clinical outcomes. As a result, visualizations based on such low-dimensional embeddings may misleadingly suggest patterns that reflect confounding variation rather than true biological or clinical differences (Fig. 1) and therefore not align with global association tests. Beyond PCoA-based approaches, several other dimension reduction techniques have been applied to high-dimensional biological data. Nonlinear methods such as t-distributed stochastic neighbor embedding (t-SNE) [21] and Uniform Manifold Approximation and Projection (UMAP) [22] have gained popularity for visualizing complex data structures, particularly in single-cell sequencing studies [23, 24]. However, both methods remain unsupervised and do not adjust for covariates, limiting their utility for revealing outcome-relevant patterns independent of nuisance variation.

**Figure 1:**
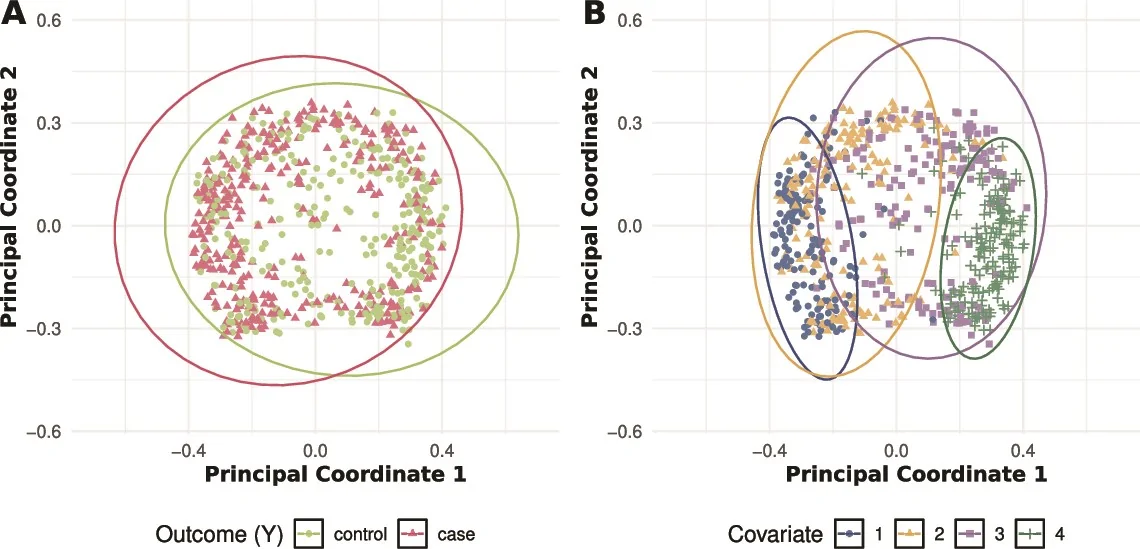
PC biplots from simulated microbiome profiling data. Axes show the first two PCs explaining the most variation. (A) Biplot colored by a binary outcome. (B) Biplot colored by a categorical nuisance covariate. Both variables are associated with the microbiome. There is only a clear pattern among samples based upon covariate level and not by case-control status, illustrating how PCoA can fail to prioritize true outcome differences.

Moreover, if these unsupervised dimensions are used as predictors in downstream regression or classification analyses, the models may prioritize variation driven by nuisance factors, reducing predictive accuracy for the outcomes of interest. Recent advances in PC(o)A-based approaches have focused on simultaneous covariate adjustment in dimensionality reduction. Lin *et al*. [25] proposed acPCA, a penalized PCA framework that removes confounding variation by minimizing the dependence between principal components and observed covariates. Building on this, Wang *et al*. [26] introduced acPCoA, which extends covariate adjustment to settings where only pairwise non-Euclidean distances are available to describe the samples. By integrating PCoA with a penalty on covariate association, acPCoA enables covariate adjusted dimension reduction directly from distance matrices. However, both of these methods remain unsupervised and may fail to prioritize features or directions related to the outcome in predictive models. Related distance-based constrained ordination methods, including distance-based redundancy analysis (db-RDA) and canonical analysis of PCs, use explanatory variables or prespecified groups to partition or discriminate variation in a dissimilarity matrix, but are primarily designed for explanatory ordination or hypothesis testing rather than outcome prediction for new samples [27]. Outcome-supervised dimension reduction methods such as partial least squares (PLS) and its discriminant analysis variant (PLS-DA) [28, 29] explicitly incorporate outcome information by constructing projections that maximize covariation between features and the response. While PLS has been extended to accommodate nonlinear relationships and sparsity constraints [30, 31], these methods do not account for covariates. Recently, PeDecURe [32] extended PLS to handle simultaneous covariate adjustment, but operates only on Euclidean distances and thus is not optimized for data that are not approximately normally distributed, such as microbiome profiling data.

Accordingly, we propose a supervised extension of acPCoA, referred to as supervised and covariate-adjusted principal coordinates analysis (scaPCoA), that integrates outcome information into the learning of low-dimensional embeddings, while simultaneously removing variation attributable to nuisance covariates. Unlike methods that are restricted to Euclidean distances, our approach directly operates on any distance matrix, extending applicability to more data types, including microbiome data and beyond. By constructing projections that emphasize outcome-relevant variation orthogonal to nuisance structures, the resulting coordinates facilitate improved predictive performance, as well as visualizations that correspond to global association tests such as MiRKAT [20]. The remainder of the paper is organized as follows. First, we introduce our proposed scaPCoA algorithm and estimation procedure. Second, we introduce a new approach to define low-dimensional embeddings for new, unseen samples using the projections learned from training data. Third, we evaluate the method’s performance via simulation studies and applications to real-world microbiome profiling data.

## Materials and methods

Before detailing our proposed supervised and covariate-adjusted method, scaPCoA, and a modification to incorporate further residualization of covariate effects (scaPCoA-R), we first review the procedures for PCoA and acPCoA. Let $\boldsymbol{y}$ denotes the *n × 1* outcome vector, where *n* is the number of samples. Let $\boldsymbol{X}$ denotes the *n × p* microbial feature matrix, where *p* is the number of microbial features, and $x_{ik}$ denotes the number of sequencing reads for the *k*th microbe in the *i*th sample. Let $\boldsymbol{Z}$ denotes the *n × q* nuisance variable matrix, where *q* is the number of covariates and $z_{il}$ denotes the value of the *l*th nuisance covariate for the *i*th sample. We assume that the covariates represented by the columns in $\boldsymbol{Z}$ are correlated with the microbial features represented by the columns in $\boldsymbol{X}$ and potentially the outcome vector $\boldsymbol{y}$. Let $\boldsymbol{D}$ denotes an *n × n* sample pairwise distance matrix of $\boldsymbol{X}$, where $d_{ij}$ is the distance between samples *i* and *j*. The choice of distance metric depends on the data type and the types of associations to prioritize (e.g. abundance, presence–absence, or phylogenetic). Common choices include Bray–Curtis (BC) dissimilarity, weighted and unweighted UniFrac distance, Aitchison distance, and Jaccard index. We let $\boldsymbol{D}$ be the BC dissimilarity for the demonstration in this paper. Other distances can be applied similarly.

### Principal coordinates analysis

PCoA is considered a generalization of PCA. The objective of PCA is to find directions of maximum variance under the assumption of Euclidean geometry, which is equivalent to solving the following optimization problem sequentially for *t = 1, … , m*, where *m < \min (n,p)*: $\underset{\boldsymbol{v}_{t}}{\arg \max } \; \boldsymbol{v}_{t}^\top \boldsymbol{X}^\top \boldsymbol{X} \boldsymbol{v}_{t}$, subject to $\|\boldsymbol{v}_{t}\|_{2}^{2} \leq 1, \boldsymbol{v}_{t} \perp \boldsymbol{v}_{t^{\prime}} \text{ for}\ t^{\prime} = 1, \dots , t-1$. The orthogonality constraint on $\boldsymbol{v}_{t}$ ensures that successive directions are uncorrelated, yielding a set of interpretable and non-redundant components. The resulting estimated $\hat{\boldsymbol{v}}_{1}, \dots , \hat{\boldsymbol{v}}_{m}$ are collected in the loading matrix $\hat{\boldsymbol{V}} = [\hat{\boldsymbol{v}}_{1}, \dots , \hat{\boldsymbol{v}}_{m}] \in \mathbb{R}^{p \times m}$, which are equivalent to the eigenvectors of $\boldsymbol{X}^\top \boldsymbol{X}$. Thus, PCA can be performed either by solving the optimization problem or via eigendecomposition of $\boldsymbol{X}^\top \boldsymbol{X}$. Projecting the data onto these eigenvectors gives the low-dimensional embedding $\breve{\boldsymbol{X}} = \boldsymbol{X}\hat{\boldsymbol{V}}$.

However, for many data types, including microbiome data which reflect sparse sequencing counts, the Euclidean distance is often an inappropriate or suboptimal metric. PCoA generalizes PCA by operating on non-Euclidean distance or dissimilarity matrices. The procedure consists of the following steps:

**Step 1:** Compute the pairwise distance matrix $\boldsymbol{D} \in \mathbb{R}^{n \times n}$ from $\boldsymbol{X}$.
**Step 2:** Convert $\boldsymbol{D}$ to a symmetric Gower’s centered similarity matrix $\boldsymbol{A} = -\frac{1}{2}(\boldsymbol{I}_{n} - \frac{1}{n}\boldsymbol{J}_{n})\boldsymbol{D}^{2} (\boldsymbol{I}_{n} - \frac{1}{n}\boldsymbol{J}_{n}) \in \mathbb{R}^{n \times n}$, where $\boldsymbol{I}_{n}$ is an *n × n* identity matrix and $\boldsymbol{J}_{n}$ is an *n × n* matrix of ones.
**Step 3:** Perform eigendecomposition of $\boldsymbol{A} = \boldsymbol{Q}\boldsymbol{\Theta } \boldsymbol{Q}^{T}$, where $\boldsymbol{\Theta } = \mathrm{diag}(\theta _{1}, \dots , \theta _{m}), m < n,$ contains the nonnegative eigenvalues above a small threshold, and $\boldsymbol{Q} \in \mathbb{R}^{n \times m}$ contains the corresponding eigenvectors. Only eigenvectors with positive eigenvalues are retained to ensure non-imaginary axes.
**Step 4:** Construct $\hat{\boldsymbol{X}}=\boldsymbol{Q}\boldsymbol{\Theta }^{\frac{1}{2}}$, a low-dimensional embedding of the samples in the PC space.

In this way, PCoA can be interpreted as extending the eigendecomposition framework of PCA to accommodate more generalized notions of pairwise dissimilarity. When Euclidean distances are used, the two methods are equivalent. Importantly, by operating on distance matrices rather than the original data, PCoA can capture nonlinear relationships in the data through the choice of distance metric. Henceforth, we denote the PCoA embedding by $\hat{\boldsymbol{X}} = [\hat{\boldsymbol{x}}_{1}, \dots , \hat{\boldsymbol{x}}_{m}] = \boldsymbol{Q}\boldsymbol{\Theta }^{1/2}$. This quantity is the final embedding produced by standard PCoA and serves as the input embedding for acPCoA (Section Adjustment for confounding principal coordinates analysis), scaPCoA (Section A supervised and covariate adjusted PCoA algorithm), and scaPCoA-R (Section A supervised and covariate adjusted PCoA algorithm with residualization).

### Adjustment for confounding principal coordinates analysis

The acPCoA algorithm [26] performs unsupervised dimensionality reduction while simultaneously adjusting for nuisance covariates. The algorithm’s first step is to obtain the PCs, $\hat{\boldsymbol{X}}$, from the PCoA procedure described in Section Principal coordinates analysis. Next, we solve the following optimization problem for *t=1,… , m*, where *m < n*:


(1) \begin{align*}& \underset{\boldsymbol{v}_{t}}{\arg\max} \; \boldsymbol{v}_{t}^{T} \boldsymbol{\hat{X}}^{T} \boldsymbol{\hat{X}} \boldsymbol{v}_{t} - \lambda \boldsymbol{v}_{t}^{T} \boldsymbol{\hat{X}}^{T} \boldsymbol{Z}\boldsymbol{Z}^{T} \boldsymbol{\hat{X}} \boldsymbol{v}_{t} \nonumber \\ &\quad\mathrm{s.t.} \|\boldsymbol{v}_{t}\|_{2}^{2} \leq 1, \boldsymbol{v}_{t} \perp \boldsymbol{v}_{t^{\prime}} \text{ for}\ t^{\prime} = 1, \dots, t-1.\end{align*}

The eigenvectors are assembled into the matrix $\hat{\boldsymbol{V}} = [\hat{\boldsymbol{v}}_{1}, \dots , \hat{\boldsymbol{v}}_{m}] \in \mathbb{R}^{m\times m}$. The first term encourages projections that preserve the overall variance captured by $\hat{\boldsymbol{X}}$, while the second term penalizes projections that align with the nuisance covariates in $\boldsymbol{Z}$. The tuning parameter *λ * controls the strength of the penalty against the nuisance covariates. When * λ = 0 *, there is no adjustment for $ \boldsymbol{Z} $. As $ \lambda \to \infty $, the solution approaches a subspace of $ \hat{\boldsymbol{X}} $ that is orthogonal to $ \boldsymbol{Z}$. The learned embedding is obtained by projecting the PCoA coordinates onto the new estimated directions: $\breve{\boldsymbol{X}} = \hat{\boldsymbol{X}} \hat{\boldsymbol{V}}$.

### A supervised and covariate adjusted PCoA algorithm

Our approach focuses on the extension to incorporating outcome supervision. This enables the identification of principal directions of the microbial data that are associated with the outcome $\boldsymbol{y}$, while adjusting for nuisance covariates $\boldsymbol{Z}$. Similar to acPCoA, we first compute $\hat{\boldsymbol{X}}$ via the standard PCoA procedure (Section Principal coordinates analysis). Next, we specifically seek linear combinations of the original PCs, $\boldsymbol{\hat{X}}$, that maximize the covariance between $\boldsymbol{\hat{X}}$ and $\boldsymbol{y}$ and simultaneously penalize directions aligned with nuisance variation explained by $\boldsymbol{Z}$. Accordingly, we solve the following optimization problem for *t=1,… , m*, where *m < n*:


(2) \begin{align*}& \underset{\boldsymbol{v}_{t}}{\arg\max} \; \boldsymbol{v}_{t}^{T} \boldsymbol{\hat{X}}^{T} \boldsymbol{y} \boldsymbol{y}^{T} \boldsymbol{\hat{X}} \boldsymbol{v}_{t} - \lambda \boldsymbol{v}_{t}^{T} \boldsymbol{\hat{X}}^{T} \boldsymbol{Z} \boldsymbol{Z}^{T} \boldsymbol{\hat{X}} \boldsymbol{v}_{t} \nonumber \\ &\quad\mathrm{s.t.} \|\boldsymbol{v}_{t}\|_{2}^{2} \leq 1, \boldsymbol{v}_{t} \perp \boldsymbol{v}_{t^{\prime}} \text{ for}\ t^{\prime} = 1, \dots, t-1.\end{align*}

The eigenvectors are assembled into the matrix $\hat{\boldsymbol{V}} = [\hat{\boldsymbol{v}}_{1}, \dots , \hat{\boldsymbol{v}}_{m}] \in \mathbb{R}^{m \times m}$. In our proposed objective function, the first term encourages the projection direction, $\boldsymbol{v}_{t}$, to align with variation in $\hat{\boldsymbol{X}}$ that is related to $\boldsymbol{y}$, effectively maximizing their shared covariance. The second term imposes a penalty on directions that align with variation explained by nuisance variables $\boldsymbol{Z}$. This regularization discourages the inclusion of nuisance variation in the learned components. The supervised embedding is defined as $\breve{\boldsymbol{X}} = \hat{\boldsymbol{X}} \hat{\boldsymbol{V}}$. The tuning parameter *λ * controls the strength of the nuisance variation adjustment. Note that when *λ = 0*, the objective reduces to a PLS type objective, with supervision but no covariate adjustment. A key difference, however, is that standard PLS would use $\boldsymbol{X}$, the original features, directly instead of $\hat{\boldsymbol{X}}$ as in Equation (2).

### A supervised and covariate adjusted PCoA algorithm with residualization

When interdependence exists among $\boldsymbol{X}$, $\boldsymbol{y}$, and $\boldsymbol{Z}$, the projected directions obtained from solving Equation (2) may still retain residual associations with $\boldsymbol{Z}$ or exhibit a weakened association with the outcome $\boldsymbol{y}$. To address this, we further refine our proposed scaPCoA procedure with an additional univariate residualization step. We refer to this procedure as scaPCoA-R. This approach is a generalization of the PeDecURe procedure from Weinstein *et al*. [32] to any distance metric. When the Euclidean distance is used, the two are similar. The steps are as follows:

**Step 1:** Obtain $ \hat{\boldsymbol{X}}$ from the PCoA procedure (Section Principal coordinates analysis).
**Step 2:** Fit a linear model for each PCoA coordinate $\hat{\boldsymbol{x}}_{t}$, for *t = 1,… ,m* where *m < n*: $\hat{\boldsymbol{x}}_{t} = \boldsymbol{1}\beta _{0}^{(t)} +\boldsymbol{y} \beta _{y}^{(t)}+ \boldsymbol{Z} \boldsymbol{\beta }_{Z}^{(t)} + \boldsymbol{\epsilon }$, $\boldsymbol{\epsilon } \sim N(0, \sigma ^{2})$. Obtain principal direction-specific: intercept $\hat{\beta }_{0}^{(t)}$, effect of $\boldsymbol{y}$ (conditional on $\boldsymbol{Z}$) $\hat{\boldsymbol{\beta }}_{y}^{(t)}$, and effect of $\boldsymbol{Z}$ (conditional on $\boldsymbol{y}$) $\hat{\boldsymbol{\beta }_{Z}}^{(t)}$.
**Step 3:** Identify two sets of partial residuals, $\boldsymbol{X}^{*}=[\boldsymbol{x}_{1}^{*}, \ldots , \boldsymbol{x}_{m}^{*}]$ and $\tilde{\boldsymbol{X}}=[\tilde{\boldsymbol{x}}_{1}, \ldots , \tilde{\boldsymbol{x}}_{m}]$, where $\boldsymbol{x}^{*}_{t} = \hat{\boldsymbol{x}}_{t} - \boldsymbol{Z}\hat{\boldsymbol{\beta }}_{Z}^{(t)}$ and $\tilde{\boldsymbol{x}}_{t} = \hat{\boldsymbol{x}}_{t} - \boldsymbol{y}\hat{\boldsymbol{\beta }}_{y}^{(t)}$, for *t = 1, … , m*.
**Step 4:** Estimate $\hat{\boldsymbol V} = [\hat{\boldsymbol v}_{1},\dots ,\hat{\boldsymbol v}_{m}]$ by solving the following optimization problem, for *t=1,… , m*, where *m < n*:
  (3) \begin{align*} &\underset{\boldsymbol{v}_{t}}{\arg\max} \; \boldsymbol{v}_{t}^{T} \boldsymbol{X}^{*^{T}}\boldsymbol{y}\boldsymbol{y}^{T}\boldsymbol{X}^{*}\boldsymbol{v}_{t} - \lambda \boldsymbol{v}_{t}^{T}\tilde{\boldsymbol{X}}^{T}\boldsymbol{Z}\boldsymbol{Z}^{T}\tilde{\boldsymbol{X}}\boldsymbol{v}_{t} \; \mathrm{s.t.} \|\boldsymbol{v}_{t}\|_{2}^{2} \nonumber \\ &\quad \leq 1, \boldsymbol{v}_{t} \perp \boldsymbol{v}_{t^{\prime}} \text{ for}\ t^{\prime} = 1, \dots, t-1.\end{align*}

By using the residualized matrix $\boldsymbol{X}^{*}$ in the first term of Equation (3), we mitigate the risk of overemphasizing directions in which the outcome $\boldsymbol{y}$ is spuriously associated with the nuisance covariates $\boldsymbol{Z}$. Furthermore, using $\tilde{\boldsymbol{X}}$ in the penalty term prevents the overpenalization of directions where $\boldsymbol{Z}$ genuinely shares a signal with $\boldsymbol{y}$. The learned supervised embedding is $\breve{\boldsymbol{X}} = \hat{\boldsymbol{X}} \hat{\boldsymbol{V}}$.

### Method evaluation using partial correlation

We use the partial correlation between the PCs, $\boldsymbol{Z}$ and $\boldsymbol{y}$ to evaluate the method performance. We denote the *t*th estimated PC by $\breve{\boldsymbol{x}}_{t}$, *t = 1, … ,m*. For each $\breve{\boldsymbol{x}}_{t}$, we first compute the pairwise correlation among $\breve{\boldsymbol{x}}_{t}, \boldsymbol{Z}$, and $ \boldsymbol{y}$, and collect them into a correlation matrix $\boldsymbol{\Omega }_{t}$. The type of correlation is chosen adaptively; if both variables are binary, we use Matthews correlation coefficient; if one variable is continuous and the other is binary, we use the point-biserial correlation coefficient; otherwise, we use Pearson correlation. Based on this, we utilize Pearson correlation for categorical covariates with more than two groups. We then compute the precision matrix $\boldsymbol{H}_{t} = \boldsymbol{\Omega }^{-1}_{t}$. If $ \boldsymbol{\Omega }_{t}$ is singular, we instead use the Moore–Penrose pseudoinverse. Partial correlations are then obtained from the entries of $\boldsymbol{H}_{t}$. Specifically, let $\boldsymbol{h}_{u,v}$ denotes the (*u,v*) entry of $\boldsymbol{H}_{t}$, the partial correlation between $\breve{\boldsymbol{x}}_{t}$ and $\boldsymbol{y}$ given $\boldsymbol{Z}$ is pcor$(\breve{\boldsymbol{x}}_{t}, \boldsymbol{y}|\boldsymbol{Z}) = -\frac{h_{\boldsymbol{y}, \breve{\boldsymbol{x}}_{t}}}{\sqrt{h_{\boldsymbol{y},\boldsymbol{y}} \cdot h_{\breve{\boldsymbol{x}}_{t},\breve{\boldsymbol{x}}_{t}}}}$, and the partial correlation between $\breve{\boldsymbol{x}}_{t}$ and $\boldsymbol{z}_{l}$ given all other variables is pcor$(\breve{\boldsymbol{x}}_{t}, \boldsymbol{z}_{l}|\boldsymbol{y}, \boldsymbol{Z}_{-l}) = -\frac{h_{\boldsymbol{z}_{l}, \breve{\boldsymbol{x}}_{t}}}{\sqrt{h_{\boldsymbol{z}_{l},\boldsymbol{z}_{l}} \cdot h_{\breve{\boldsymbol{x}}_{t},\breve{\boldsymbol{x}}_{t}}}}$.

### Selecting optimal tuning parameter *λ *

The tuning parameter *λ * controls the strength of nuisance variation adjustment in acPCoA and scaPCoA(-R). An appropriate choice of *λ * balances penalizing associations with nuisance covariates $\boldsymbol{Z}$, while preserving signals relevant to the outcome $\boldsymbol{y}$. We modify a previously proposed tuning strategy [32] to incorporate an adaptive partial correlation measure that accommodates both binary and continuous outcomes and covariates, as detailed in Section Method evaluation using partial correlation. We select *λ * based on its ability to separate outcome-related and covariate-related variation in the PCs. Specifically, we choose the value of *λ * that maximizes the difference between the absolute partial correlation of each PC with outcome $\boldsymbol{y}$ (adjusting for each $\boldsymbol{z}_{l}$) and the absolute partial correlation of each PC with nuisance variables $\boldsymbol{Z}$ (adjusting for $\boldsymbol{y}$). We select the optimal *λ * from a predefined tuning grid by solving:


(4) \begin{align*} \lambda_{\mathrm{optimal}} =& \arg\max_\lambda \frac{1}{q} \sum_{l=1}^{q} \sum_{t=1}^{m} \frac{\theta_{t}}{\sum_{w=1}^{m} \theta_{w}} \nonumber \\&\left( \left| \mathrm{pcor}( \breve{\boldsymbol{x}}_{t}^{(\lambda)}, \boldsymbol{y} \mid \boldsymbol{z}_{l} ) \right| - \left| \mathrm{pcor}(\breve{\boldsymbol{x}}_{t}^{(\lambda)}, \boldsymbol{z}_{l} \mid \boldsymbol{y} ) \right| \right),\end{align*}

where $\breve{\boldsymbol{x}}_{t}^{(\lambda )}$ denotes the *t*th estimated PC when the tuning parameter is set to *λ *, *t = 1,… m*, and $\theta _{t}$ is the eigenvalue corresponding to $\breve{\boldsymbol{x}}_{t}$. The optimal value of *λ * was selected from five-fold cross-validation.

### Out-of-sample embedding procedure

To evaluate the generalizability of the proposed supervised dimension reduction method, we define an out-of-sample embedding by projecting each new sample into the low-dimensional space learned from the training data. Unlike PCA, where a linear mapping from the feature space to the principal component space enables direct projection of new observations, PCoA-based methods lack an explicit linear transformation. Instead, the embedding is constructed from pairwise distances and eigendecomposition, introducing nonlinearity and making out-of-sample extensions nontrivial. This extension is essential for a realistic assessment of model performance and ensures that the dimension reduction procedure remains applicable in scenarios where new data become available after the initial embedding is constructed.

We obtain training samples $\boldsymbol{X}_{\mathrm{train}} \in \mathbb{R}^{n_{1} \times p}$ and out-of-sample data $\boldsymbol{X}_{\mathrm{test}} \in \mathbb{R}^{n_{2} \times p}$. We perform the PCoA procedure described in Section Principal coordinates analysis on the training data to obtain $\boldsymbol{D}_{\mathrm{train}}$ and $\boldsymbol{A}_{\mathrm{train}} \in \mathbb{R}^{n_{1} \times n_{1}}$, which are used to estimate $\hat{\boldsymbol{X}}_{\mathrm{train}} \in \mathbb{R}^{n_{1} \times m}$. We then estimate a projection matrix $\hat{\boldsymbol{V}} \in \mathbb{R}^{m \times m}$, under a decomposition objective from acPCoA (1), scaPCoA (2), or scaPCoA-R (3). The projected PCs for the training set are then computed as $\breve{\boldsymbol{X}}_{\mathrm{train}} = \hat{\boldsymbol{X}}_{\mathrm{train}} \hat{\boldsymbol{V}} \in \mathbb{R}^{n_{1} \times m}$.

To embed each new sample in the same low-dimensional space as the training data, we first compute the pairwise distance matrix $\boldsymbol{D}_{\mathrm{train,test}} \in \mathbb{R}^{n_{1} \times n_{2}}$ and the Gower-centered similarity matrix $\boldsymbol{A}_{\mathrm{train,test}} \in \mathbb{R}^{n_{1} \times n_{2}}$. Let $\boldsymbol{a}_{g} \in \mathbb{R}^{n_{1} \times 1}$ denotes the *g*th column of $\boldsymbol{A}_{\mathrm{train,test}}$, which indicates the similarity between the *g*th new sample and each training point. Then, the Trosset out-of-sample embedding [33] for the *g*th new sample is defined as: $\hat{\boldsymbol{u}}_{g} = \underset{\boldsymbol{u}}{\arg \min } \left \{ 2\sum _{i=1}^{n_{1}} (a_{gi} -\hat{\boldsymbol{x}}_{i}^\top \boldsymbol{u})^{2} + (\boldsymbol{\beta }_{g} - \boldsymbol{u}^\top \boldsymbol{u})^{2} \right \}$, where $\hat{\boldsymbol{x}}_{i}$ is the PCoA embedding of the *i*th training sample and $\boldsymbol{\beta }_{g} = \frac{1}{2n_{1}}\sum _{i=1}^{n_{1}} a_{gi}$ is an estimate of the squared norm of the *g*th new sample in the centered inner-product space. This optimization is solved for each test set sample to yield the matrix $\hat{\boldsymbol{X}}_{\mathrm{test}} = [\hat{\boldsymbol{u}}_{1}, \dots , \hat{\boldsymbol{u}}_{n_{2}}]^\top \in \mathbb{R}^{n_{2} \times m}$. Finally, we compute the out-of-sample embedding as $\breve{\boldsymbol{X}}_{\mathrm{test}} = \hat{\boldsymbol{X}}_{\mathrm{test}} \hat{\boldsymbol{V}} \in \mathbb{R}^{n_{2} \times m}$. This procedure allows us to project out-of-sample data using the projection learned in training, without recomputing the embedding with combined data. This approach can be used with data splitting in regression and classification tasks to avoid data double dipping.

## Results

### Simulation studies

In simulation, let the microbiome data ($\boldsymbol{X} \in \mathbb{R}^{n \times p}$) be comprised of *p = 200* features from $n\in \{50,100,200\}$ samples, along with *q = 3* covariates defined as follows: $ \boldsymbol{z}_{1}, \boldsymbol{z}_{2} \overset{iid}{\sim } \mathrm{Normal}(0, 1)$, with $\mathrm{Corr}(\boldsymbol{z_{1}}, \boldsymbol{z_{2}}) = 0.4$, and $\boldsymbol{z}_{3} \overset{iid}{\sim } \mathrm{Bernoulli}(0.3)$. Let $\boldsymbol{Z} = [\boldsymbol{z}_{1}, \boldsymbol{z}_{2}, \boldsymbol{z}_{3}] \in \mathbb{R}^{n \times 3}$. We first simulated $\boldsymbol{X}^{\circ }$ from a zero-inflated latent Dirichlet allocation model [34]. The total number of sequencing reads per sample was drawn from a discrete uniform distribution with bounds (1000, 30 000). The model’s hyperparameters were set as *α * = 0.1, *π =0.7*, *a=0.6*, *b=10*, and the number of latent factors was set to five, which is the default specification suggested by Deek and Li [34]. To introduce realistic covariate effects, we modified a randomly selected 50% of the microbial features to be associated with the covariates $\boldsymbol{Z}$. For each of the selected features $\boldsymbol{x}_{k}^{\circ }$, we generated the covariate-associated feature $\boldsymbol{x}_{k}$ from a Poisson distribution with mean $\mu _{ik} = \{\mathrm{exp}( \boldsymbol{z}_{i}^{\top }\boldsymbol{\gamma })\}\boldsymbol{x}_{ik}^{\circ }$ for a sample *i*, where $\boldsymbol{\gamma } = [0.5, -0.5, 0.5]^\top $. We used this formulation to ensure that the new $\boldsymbol{x}_{k}$ features maintained variance and overdispersion levels similar to those of the original variables. Moreover, this multiplicative formulation ensured that the initial zero counts remained zero, maintaining the same zero-inflated structure. For the unselected microbes, we set $\boldsymbol{x}_{k}=\boldsymbol{x}_{k}^{\circ }$. We denote the resulting covariate-associated microbial count matrix as $\boldsymbol{X}$, which is the microbial feature matrix used going forward.

To construct the outcome $\boldsymbol{y}$ from the microbial counts $\boldsymbol{X}$ and covariates $\boldsymbol{Z}$, we proceeded as follows. First, we selected the 15 microbial features with the highest correlation with one another, denoted as $\{{x}_{i1}, \dots , {x}_{i15}\}$, where $x_{ik}$ denotes the *k*th microbial feature for the *i*th sample, for *k=1,… ,15*. Next, we defined the linear predictor for sample *i*: $f(\boldsymbol{x}_{i}, \boldsymbol{z}_{i}) = 0.005\sum _{k=1}^{15} x_{ik} + z_{i1} - z_{i2} + z_{i1}z_{i3} - \mathrm{offset}.$ For a binary outcome, we then set $\pi _{i} = \mathrm{expit}(f(\boldsymbol{x}_{i}, \boldsymbol{z}_{i}))$, where $\pi _{i}$ is the probability of an event occurring (e.g. disease occurrence). The offset was chosen as the median of $\{0.005 \sum _{k=1}^{15} x_{ik} + z_{i1} - z_{i2} + z_{i1}z_{i3}\}_{i=1}^{n}$, which centered the linear predictor around zero, and ensured that $\pi _{i}$ yielded an approximately balanced proportion of binary outcomes. Finally, we assumed $y_{i} \sim \mathrm{Bernoulli}(\pi _{i})$. For continuous outcomes, we let $y_{i} = \frac{f(\boldsymbol{x}_{i}, \boldsymbol{z}_{i}) - \frac{1}{n}\sum _{i=1}^{n}f(\boldsymbol{x}_{i}, \boldsymbol{z}_{i})} {\mathrm{SD}[f(\boldsymbol{x}_{i}, \boldsymbol{z}_{i})]} + \epsilon _{y_{i}}$, where $\epsilon _{y_{i}} \sim N(0,\,0.5^{2})$. For each outcome setting, we repeated the simulation for 100 iterations. As an additional negative-control analysis, we considered a null setting in which the outcome was generated independently of both the microbial features and covariates (Supplementary Section S1).

For visualization tasks, we utilized all *n* samples within each sample size setting. For prediction, we evaluated out-of-sample performance by randomly partitioning each simulated dataset into $n_{1} = 0.7n$ training samples and $n_{2} = 0.3n$ held-out test samples. We retained the top three PCs (i.e. *m = 3*), therefore yielding a $\hat{\boldsymbol{V}} \in \mathbb{R}^{3 \times 3}$. This results in estimated PCs $\breve{\boldsymbol{X}} \in \mathbb{R}^{n \times 3}$ for visualization and $\breve{\boldsymbol{X}}_{\mathrm{train}} \in \mathbb{R}^{n_{1} \times 3} $ and $ \breve{\boldsymbol{X}}_{\mathrm{test}} \in \mathbb{R}^{n_{2} \times 3}$ for prediction. Additionally, we conducted simulations comparing the Trosset out-of-sample procedure with full PCoA re-embedding of the combined training and test data, which showed that the proposed out-of-sample procedure resulted in only minimal additional train-test distance reconstruction error under matched training and testing distributions (Supplementary Section S2).

We evaluated performance through both visualization and predictive modeling. For visualization, we assessed how well each method separates outcomes in the PC space. For prediction, we first examined the partial correlations of the estimated PCs with both the outcome and covariates in training and out-of-sample data (Section Method evaluation using partial correlation). To further evaluate the ability of the estimated PCs to capture outcome-relevant variation, we fit generalized linear models regressing the outcome on the PCs, both with and without covariate adjustment. We quantified predictive performance using area under the receiver operating characteristic curve (AUC) for binary outcomes and root mean square error (RMSE) for continuous outcomes, computed on the held-out test set. We additionally compared the PCoA-based methods with three full-feature machine learning (ML) approaches: LASSO, Random Forest, and XGBoost. These models were fit using all clr-transformed microbial features, either without or with the covariates, and were evaluated using the same held-out test samples as the PCoA-based methods.

### Simulation results

Unsupervised PCoA and acPCoA failed to capture outcome-relevant variations, as expected, since their embeddings were driven solely by the overall distance structure, without reference to the outcome of interest. In contrast, supervised methods scaPCoA and scaPCoA-R incorporated outcome information during projection, enabling enhanced visual discrimination between groups. Figure 2A/B demonstrates this advantage, the supervised methods showed a clear separation between binary outcome groups and a gradient for continuous outcomes along the first PC axis. Silhouette widths range from *-1* to *1* and indicate how well matched a point is to its neighbors. Silhouette scores are the average of the widths, with larger positive values indicating greater separation between groups. Consistent with the visual pattern, the silhouette scores were near zero for PCoA and acPCoA (0.024 and 0.021, respectively), but substantially higher for scaPCoA and scaPCoA-R (0.339 and 0.403), indicating greater outcome-group separation in the supervised embeddings. Moreover, the PCA-based methods, unsupervised PCA and supervised PeDecURe, (Supplementary Fig. S3A/B) and nonlinear unsupervised methods, t-SNE and UMAP (Supplementary Fig. S4A/B) also did not show any clear separation. These results indicate the supervised PCoA embeddings are more effective in capturing outcome-relevant microbial differences. The visualizations are presented for *n=100* and similar patterns were observed for *n=50* and *n=200*.

**Figure 2:**
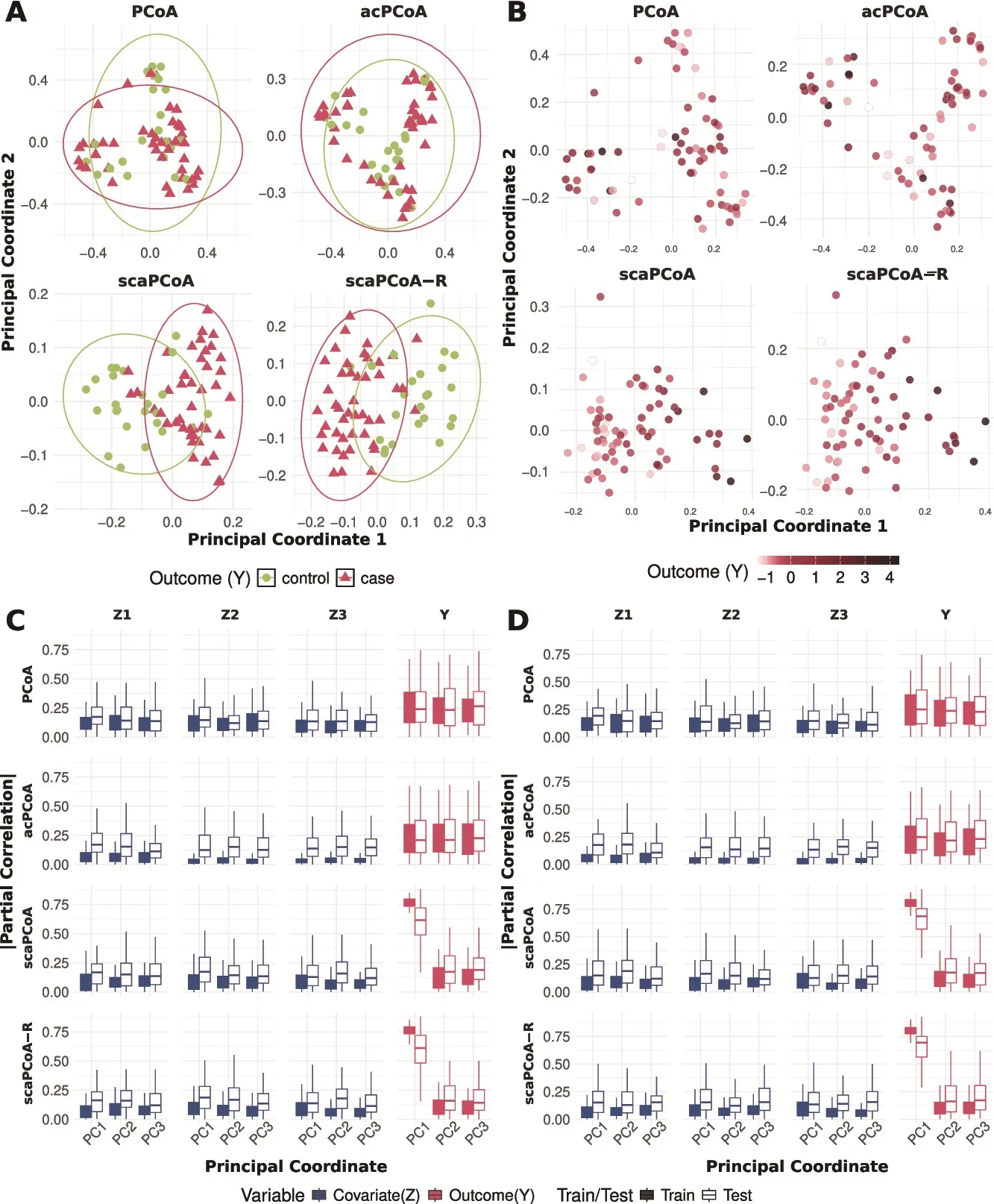
Simulation results for PCoA, acPCoA, scaPCoA, and scaPCoA-R at *n=100* and *p=200*. (A) PC biplots for a binary outcome. Axes show the first two PCs. (B) PC biplots, as described in (A), for a continuous outcome. (C) Boxplots of the absolute partial correlation between the first three estimated PCs and $\boldsymbol{y}$ (adjusting for $\boldsymbol{Z}$), and between PCs and each $\boldsymbol{Z}$ (adjusting for $\boldsymbol{y}$ and the remaining $\boldsymbol{Z}$), for a binary outcome. (D) Partial correlation boxplots, as described in (C), for a continuous outcome.

Figure 2C/D shows the absolute partial correlations of the top three estimated PCs with the covariates and the outcome for PCoA, acPCoA, scaPCoA, and scaPCoA-R. Analogous plots using PCA-based methods are shown in Supplementary Fig. S3C/D. We used the top three coordinates as a parsimonious low-dimensional representation. The first scaPCoA coordinate explained nearly all fitted-coordinate variability, and the first three captured the dominant supervised structure (Supplementary Fig. S7). The unsupervised methods (PCA, PCoA, and acPCoA) failed to identify PCs that were strongly correlated with the outcome. Specifically, the PCs estimated by PCA and PCoA exhibited similar absolute partial correlations across the covariates and the outcome, indicating a lack of clear outcome relevance in the estimated embeddings. Although acPCoA reduced covariate partial correlations during training, these correlations increased during testing, and associations with the outcome remained low in training and out-of-sample testing data. The PCA-based supervised method, PeDecURe, extracted outcome-associated coordinates in training, but failed to reproduce these results in the out-of-sample data (Fig. S3C/D). In contrast, our proposed supervised methods, scaPCoA and scaPCoA-R, effectively recovered ordinations in which PC1 is strongly correlated with the outcome, while maintaining reduced correlations with covariates in both binary and continuous outcome settings (Fig. 2C/D). Importantly, this pattern persisted in the out-of-sample testing data, where the absolute partial correlation between PC1 and the outcome remained higher than that of covariates.

For outcome prediction, Table 1 shows that scaPCoA and scaPCoA-R consistently improved predictive performance relative to the unsupervised PCoA and acPCoA methods. For binary outcomes, the proposed methods achieved the highest mean AUC across all three sample sizes, with and without covariates, outperforming LASSO, Random Forest, and XGBoost. For continuous outcomes, scaPCoA and scaPCoA-R achieved the lowest RMSE at each sample size when covariates were not included in the prediction model. When covariates were included, scaPCoA and scaPCoA-R remained optimal at *n=100* and competitive against Random Forest for *n=50* and XGBoost for *n=200*. Additionally, the results from the PCA-based methods also showed lower predictive performance (Supplementary Section S3.1) than the proposed methods, despite PeDecURe also being a supervised approach.

**Table 1:** Simulation results comparing model performance in classification [AUC; mean(SD)] and regression [RMSE; mean(SD)] tasks with *p=200* microbial features under different sample size settings.

Outcome	Method	*n=50*		*n=100*		*n=200*	
		Without Z	With Z	Without Z	With Z	Without Z	With Z
Binary	PCoA	0.815 (0.122)	0.795 (0.120)	0.733 (0.104)	0.771 (0.099)	0.778 (0.097)	0.785 (0.091)
(AUC)	acPCoA	0.701 (0.155)	0.759 (0.137)	0.716 (0.129)	0.759 (0.106)	0.725 (0.100)	0.752 (0.093)
	scaPCoA	0.835 (0.126)	0.823 (0.108)	**0.852** (0.086)	**0.860** (0.080)	0.894 (0.056)	**0.894** (0.052)
	scaPCoA-R	**0.836** (0.124)	**0.824** (0.119)	0.850 (0.097)	0.858 (0.096)	**0.896** (0.050)	0.894 (0.050)
	LASSO	0.714 (0.154)	0.724 (0.145)	0.712 (0.111)	0.727 (0.106)	0.806 (0.072)	0.807 (0.070)
	Random Forest	0.808 (0.136)	0.802 (0.138)	0.805 (0.095)	0.811 (0.093)	0.867 (0.061)	0.869 (0.060)
	XGBoost	0.747 (0.149)	0.760 (0.124)	0.784 (0.108)	0.797 (0.101)	0.866 (0.062)	0.867 (0.059)
Continuous	PCoA	0.859 (0.296)	0.829 (0.268)	1.006 (0.240)	0.981 (0.226)	0.822 (0.285)	0.823 (0.262)
(RMSE)	acPCoA	0.892 (0.298)	0.858 (0.264)	1.018 (0.239)	0.992 (0.224)	0.862 (0.287)	0.844 (0.256)
	scaPCoA	**0.811** (0.292)	0.843 (0.240)	0.860 (0.195)	0.878 (0.172)	**0.656** (0.259)	0.700 (0.236)
	scaPCoA-R	0.815 (0.290)	0.845 (0.234)	**0.858** (0.194)	**0.876** (0.174)	0.659 (0.258)	0.701 (0.235)
	LASSO	0.930 (0.318)	0.886 (0.311)	1.034 (0.245)	1.017 (0.233)	0.789 (0.256)	0.787 (0.255)
	Random Forest	0.825 (0.306)	**0.811** (0.303)	0.904 (0.227)	0.900 (0.225)	0.675 (0.270)	0.677 (0.270)
	XGBoost	0.879 (0.338)	0.850 (0.331)	0.921 (0.218)	0.906 (0.210)	0.666 (0.247)	**0.659** (0.248)

Bold values indicate model with the best AUC or RMSE for each sample size.

Lastly, we evaluated the runtime of scaPCoA on a computing node using 2 CPU cores and 100 GB of memory, with *p=200* and a fixed tuning parameter of *λ =0.1*. Across 100 simulation replicates, mean total runtime for scaPCoA was 0.006, 0.012, and 0.278 s at *n=50*, *100*, and *500*, respectively. Corresponding runtimes for scaPCoA-R were 0.021, 0.032, and 0.346 s. At *n=500*, the mean total runtime was 0.278 s for scaPCoA and 0.346 s for scaPCoA-R. At *n=500*, the PCoA embedding, residualization, and supervised optimization steps for scaPCoA-R required 0.080, 0.075, and 0.191 s, respectively.

### Application to several colorectal cancer metagenomic profiling studies

Colorectal cancer (CRC) is the second leading cause of cancer-related mortality worldwide. The human gut microbiome plays an important role in CRC development, influencing carcinogenesis, tumor progression, and response to systemic therapies [35]. As such, characterizing gut microbial patterns in CRC patients may provide insights into disease mechanisms and potential biomarkers. Building on this evidence, we demonstrated the utility of our supervised dimensionality reduction methods in disease classification by applying them to metagenomic sequencing data from stool microbiome samples collected in five case-control studies of CRC [7, 36–39]. Together, these studies encompassed 578 adults with CRC or healthy controls, with sample sizes of 107, 104, 125, 128, and 114, respectively. The study populations originated from Austria, the USA, China, France, and Germany. These datasets were obtained from the curatedMetagenomicData package, which provides consistently processed and annotated human microbiome profiles with taxonomic, functional, and clinical metadata [40]. Raw sequencing data from the original studies were uniformly processed using the MetaPhlAn2 pipeline [41] for microbial taxonomic profiling, resulting in 808 microbial features at the species level, measured in at least one of the five studies. We excluded eight patients with missing body mass index (BMI), resulting in 570 samples for analysis, and retained only the 385 microbial species that were measured in all studies. We used the Bray-Curtis dissimilarity to quantify pairwise distances between samples.

Although the preprocessing of the raw reads was consistent between datasets, differences remained in the sample collection procedures, sequencing platforms, and laboratory protocols between the original studies. Additionally, the five studies spanned countries with distinct dietary and lifestyle habits, introducing further potential variation between studies. To account for these sources of variation, we included study membership as a covariate in the covariate-adjusted PC methods, in addition to age, sex, and BMI. We aimed to identify the PCs that captured microbial features associated with CRC, while adjusting for nuisance variables. Model performance was evaluated using five-fold cross-validation on the combined dataset. In each iteration, model estimation, tuning, and dimension reduction were performed using four training folds, and predictive performance was evaluated on the remaining held-out fold, so that each participant contributed once to the out-of-fold test results.

#### Visualizing patterns of the global microbiome

To assess global microbiome structure, we examined biplot visualizations of the first two PCs (Fig. 3A), which demonstrated that the proposed supervised and covariate-adjusted methods, scaPCoA and scaPCoA-R, achieved clear separation between CRC and healthy control participants. In contrast, the unsupervised methods, PCoA and acPCoA, showed a near complete overlap between the two groups. Consistent with these visual patterns, the silhouette scores based on the first two coordinates were 0.191 for scaPCoA and 0.183 for scaPCoA-R, compared with 0.023 for PCoA and 0.009 for acPCoA. Near-zero silhouette scores for PCoA and acPCoA indicate substantial overlap, whereas the larger positive values for scaPCoA and scaPCoA-R indicate greater case-control separation. Similarly, no clear separation was observed when applying supervised and unsupervised PCA-based methods (Supplementary Fig. S5A), as well as nonlinear unsupervised t-SNE, or UMAP (Supplementary Fig. S6). To formally test the global association between microbiome composition and CRC status, while adjusting for covariates, we applied MiRKAT, a distance-based kernel machine regression method [20]. Using Bray–Curtis dissimilarity, with adjustment for age, sex, BMI, and study, MiRKAT yielded a *P*-value * <.001*, providing independent evidence for global association between microbiome composition and CRC status that is consistent with the outcome-related structure emphasized by scaPCoA and scaPCoA-R.

**Figure 3:**
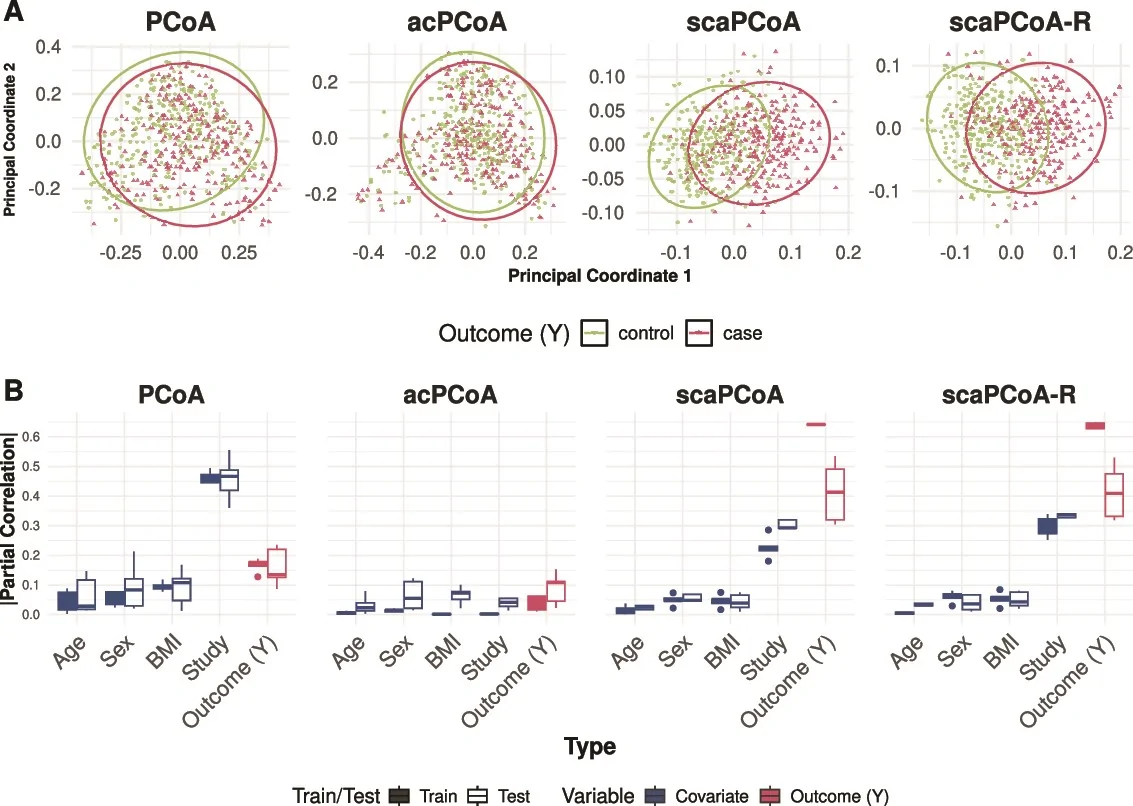
(A) PC biplots of metagenomic data from case-control CRC studies using PCoA, acPCoA, scaPCoA, and scaPCoA-R. CRC cases are represented by red triangles and healthy controls are represented by green circles. (B) Boxplots of the absolute partial correlations between the first PC and covariates or binary CRC status in training and testing data across five-fold cross-validation.

#### Prediction of colorectal cancer status

We first compared the strength of association between the first PC and CRC status across methods, quantified by partial correlations with the outcome and covariates (Fig. 3B). Unsupervised PCoA and acPCoA methods failed to extract PCs meaningfully correlated with CRC status, whereas the supervised methods, scaPCoA and scaPCoA-R succeeded. For PCoA, the first PC correlated more strongly with the study batch effect, with a median absolute partial correlation of 0.46 in testing, than with the outcome (partial correlation = 0.16). Although acPCoA reduced correlations with covariates, its association with the outcome remained weak, with a median absolute partial correlation of 0.09 in testing. In contrast, scaPCoA and scaPCoA-R methods learned a first PC that exhibited substantial association with the outcome, achieving a median absolute partial correlation of 0.41 in testing, substantially higher than those from the unsupervised methods. Although the supervised methods showed slightly higher partial correlations with the study batch effect than acPCoA, this trade-off reflects their design to enhance alignment with the outcome, which inevitably reduced the degree of covariate orthogonality. Nevertheless, for both scaPCoA and scaPCoA-R, the median absolute partial correlations with other covariates remained below 0.1 in both training and testing, while the associations with the outcome were markedly higher.

To evaluate predictive performance, we built generalized linear models using the top three estimated PCs as predictors for CRC status, with and without adjustment for age, sex, and BMI. Continuous variables age and BMI, and PCs were standardized to enable direct comparison of model coefficients. We did not include study as a predictor to prevent the model from exploiting study-specific batch effects rather than true disease-related variation. Across the five cross-validation folds, scaPCoA and scaPCoA-R achieved substantially higher AUCs than the unsupervised PCoA and acPCoA methods (Table 2), Euclidean-based PCA and PeDecURe (Supplementary Section S4.1) and LASSO (Table 2). Random Forest and XGBoost achieved the highest test AUCs, demonstrating the predictive advantage of flexible models fitted to the complete microbial feature matrix. Nevertheless, scaPCoA and scaPCoA-R retained substantial predictive information using only three estimated coordinates while additionally providing an interpretable, covariate-adjusted visualization of disease-related global microbiome variation.

**Table 2:** CRC study results comparing out-of-fold predictive performance, reported as the mean AUC (SD) across the held-out test folds from five-fold cross-validation.

Method	Without covariates	With covariates
PCoA	0.642 (0.027)	0.653 (0.063)
acPCoA	0.625 (0.030)	0.631 (0.062)
scaPCoA	0.763 (0.027)	0.763 (0.019)
scaPCoA-R	0.754 (0.029)	0.768 (0.019)
LASSO	0.714 (0.016)	0.741 (0.025)
Random Forest	0.844 (0.018)	0.853 (0.012)
XGBoost	**0.859** (0.018)	**0.862** (0.023)

Bold values indicate model with the largest AUC.

Figure 4 summarizes predictor importance for CRC classification. Importance is shown as absolute coefficients for the generalized linear models and LASSO, impurity-based importance for Random Forest, and gain for XGBoost. Values should therefore be interpreted within each method. For PCoA and acPCoA, PCs and covariates had similar coefficient magnitudes, whereas for scaPCoA and scaPCoA-R, PC1 was the dominant predictor of CRC status.

**Figure 4:**
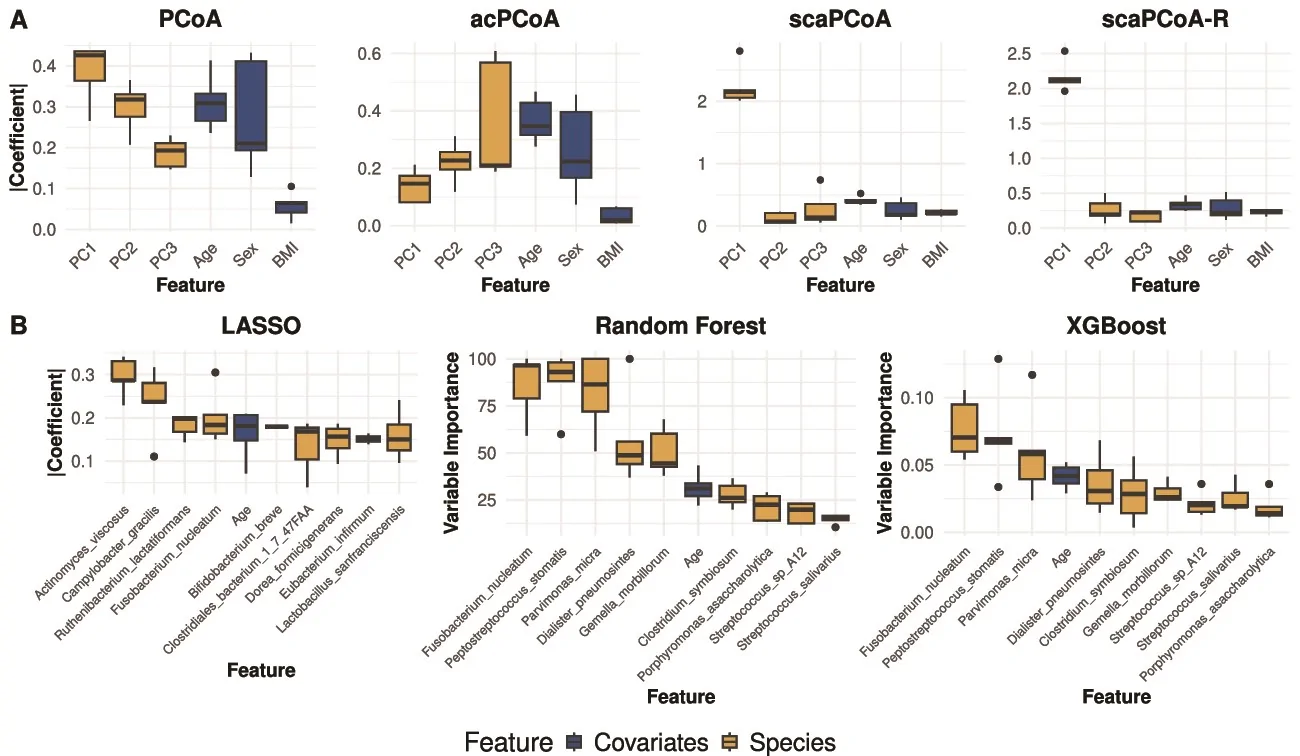
(A) Absolute coefficients from generalized linear models predicting binary CRC status using covariates and PCs estimated by PCoA, acPCoA, scaPCoA, or scaPCoA-R. (B) Feature importance for predicting binary CRC status using all microbial features and covariates, represented by absolute LASSO coefficients, Random Forest variable importance, and XGBoost gain. The ten most important features within each method are shown.

#### Microbial variable importance scores

To further identify which microbial features dominated these predictive PCs, we constructed a scaled variable importance statistic, denoted $s^{\mathrm{sum}}$, which quantifies each microbe’s contribution to the PCs (Supplementary Section S6). The microbes with the highest importance scores were then compared with those identified as important predictors from the LASSO, Random Forest, and XGBoost models (Fig. 4B). The 10 microbial features with the highest importance scores are shown in Fig. 5. The features identified by scaPCoA and scaPCoA-R differ markedly from those selected by the ML methods.

**Figure 5:**
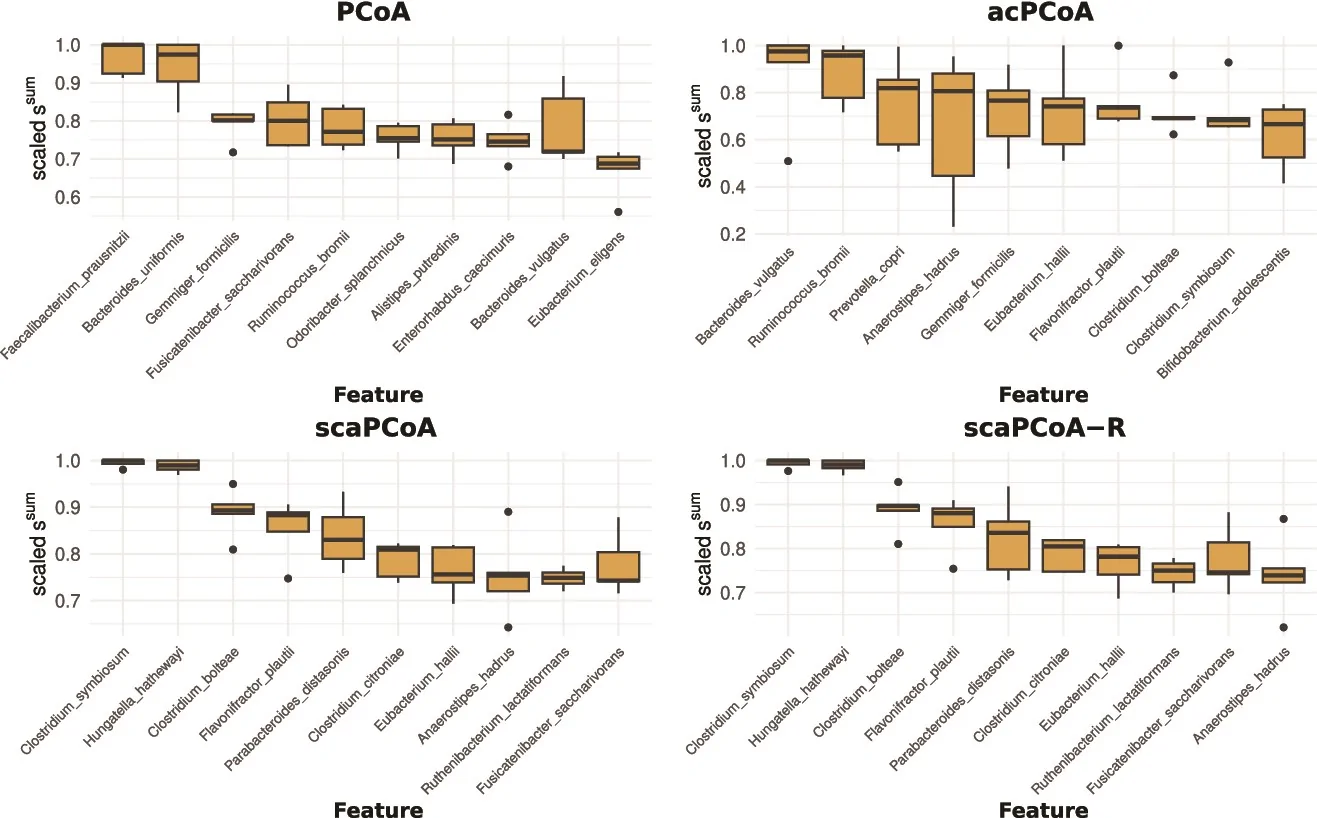
Top microbial features contributing to PCs, and therefore CRC status classification. Microbes with the highest contributions to the estimated PCs ($s^{\mathrm{sum}}$), displayed for PCoA, acPCoA, scaPCoA, and scaPCoA-R . Importance scores are rescaled between 0 and 1 within each method.

Additionally, notable differences emerged between supervised and unsupervised dimension reduction methods. Unsupervised methods (PCoA and acPCoA) predominantly identified the most abundant microbes as top contributors for CRC: *Faecalibacterium prausnitzii*, *Bacteroides uniformis*, *Bacteroides vulgatus*, and *Ruminococcus bromii*. Previous human and animal studies have shown depletion of *F. prausnitzii* and *B. uniformis* in CRC patients [42, 43], while a Mendelian randomization study linked *R. bromii* to lower CRC risk [44]. In contrast, *B. vulgatus* is elevated in CRC patients [45]. However, there is strong evidence these species are highly responsive to dietary fiber and carbohydrate consumption [46–48]. Unsupervised methods likely captured dietary variation across populations rather than CRC-specific pathogenic signals. Random Forest and XGBoost prioritized *Fusobacterium nucleatum*, *Peptostreptococcus stomatis*, *Parvimonas micra*, *Gemella morbillorum*, and other oral-associated species . Many of the previous cross-cohort studies supporting these associations analyzed the same public cohorts included in our analysis, and therefore cannot serve as independent validation [7, 8, 38].

In contrast, supervised methods scaPCoA and scaPCoA-R prioritized microbial features with biologically plausible and, in some cases, experimentally supported roles in colorectal tumorigenesis. The top-ranked features *Clostridium symbiosum*, *Hungatella hathewayi*, and *Flavonifractor plautii* were either ranked low or completely overlooked by unsupervised approaches. Although *C. symbiosum* was also identified among the top predictors by Random Forest and XGBoost, it ranked lower in those models than in the scaPCoA and scaPCoA-R feature ranking. Notably, *C. symbiosum* abundance increases stepwise along the adenoma-carcinoma sequence and, in experimental models, promotes tumorigenesis by producing branched-chain amino acids that activate mTORC1-driven cholesterol biosynthesis and downstream Sonic Hedgehog signaling [49]. *H. hathewayi* is significantly enriched in fecal microbiomes from both old- and young-onset CRC patients [50], it and has been linked to lower overall survival rates and enhanced 5-fluorouracil chemotherapy resistance [51]. Finally, *F. plautii* has been associated with CRC in young and Indian cohorts [52, 53]. Moreover, *F. plautii* has been shown to mediate the effects of environmental factors during early colorectal carcinogenesis [54].

### Application to schizophrenia microbiome profiling study

Schizophrenia is a severe psychiatric disorder with substantial clinical heterogeneity, and growing evidence suggests that alterations in the gut microbiome may contribute to disease-related immune, metabolic, and neuroactive signaling through the microbiota–gut–brain axis [55, 56]. To investigate the link between the gut microbiome and diagnosis, we applied scaPCoA and scaPCoA-R to shotgun metagenomic data from a case-control schizophrenia study [57]. We briefly describe our findings here. Compared with PCoA and acPCoA, our supervised methods produced clear separation and larger silhouette scores between schizophrenia and healthy control groups and identified PCs that were more strongly associated with schizophrenia status than with measured covariates (Supplementary Fig. S8). Using only three estimated coordinates, scaPCoA and scaPCoA-R achieved mean test AUCs of 0.929 and 0.926, respectively, which were comparable to those achieved by LASSO, Random Forest, and XGBoost models (Supplementary Table S2). Microbial importance scores highlighted *Blautia A* sp.7810 and several unclassified *Oscillospiraceae* taxa. *Blautia*-related taxa have repeatedly been associated with schizophrenia and are involved in carbohydrate fermentation and short-chain fatty acid production, while *Oscillospiraceae* taxa have also shown altered abundance in schizophrenia cohorts [57–61]. Additional details on the study data, sequencing, covariates, class balancing, prediction analysis, and microbial findings are provided in Supplementary Section S7.

## Discussion

In this paper, we propose a scaPCoA algorithm that simultaneously performs outcome-guided dimension reduction based on a general distance matrix and adjustment for nuisance covariates. Our method serves two complementary goals. First, scaPCoA derives low-dimensional embeddings that enhance the visualization of outcome-associated variation and pair well with covariate-adjusted global tests such as MiRKAT [20]. Second, it identifies predictive PCs that can be used for downstream modeling tasks. This is further enabled by an out-of-sample embedding procedure that projects new observations into the previously learned coordinate space. Consequently, when additional samples become available, their coordinates can be obtained without recomputing the PCoA embedding or refitting the scaPCoA model using the expanded dataset. This allows the learned representation to be applied to future data and facilitates its integration with regression or classification models in prospective applications. Although we focused on microbiome applications, scaPCoA is broadly applicable to other complex data structures where Euclidean-based approaches are not adequate, including other omics data.

Through both simulation studies and real data applications, we demonstrated that scaPCoA effectively recovered outcome-relevant microbial patterns while minimizing spurious associations driven by nuisance factors or technical batch effects. In visualization tasks, supervised embeddings derived from our method provided greater separation of outcome groups compared with unsupervised and PCA-based methods, making it easier to interpret disease-related patterns. In prediction tasks, scaPCoA improved out-of-sample prediction accuracy by prioritizing dimensions that encoded outcome-relevant variation orthogonal to nuisance effects. As an extension, scaPCoA-R incorporates an additional residualization step and generally achieved performance similar to that of scaPCoA. This additional residualization step may be useful when substantial interdependence exists among microbial features, covariates, and outcome, as it mitigates over-penalization and ensures that the resulting embeddings more accurately represent outcome-specific variation. Functionally, scaPCoA-R serves as an extension enhancing the method’s applicability in datasets with complex interdependency among features, covariates, and outcome. We note that this residualization assumes additive linear relationships. Therefore, scaPCoA-R may incompletely remove variation involving strong nonlinearities, interactions, or threshold effects, and consequently may underperform compared with scaPCoA in these settings. We further note that adjustment is limited to the measured covariates, and confounding from unmeasured factors may remain. Together, these properties establish scaPCoA as a versatile tool for both visualization and predictive modeling in microbiome research.

Our results from real data show that our outcome-driven approach enables the identification of biologically meaningful microbial features related to CRC status that are overlooked by unsupervised methods dominated by highly abundant or lifestyle-related taxa. scaPCoA and scaPCoA-R highlighted taxa with potential mechanistic relevance to colorectal tumorigenesis, despite full-feature ML models achieving higher predictive accuracy. In our schizophrenia application, scaPCoA and scaPCoA-R performed comparably to traditional ML methods. For translational applications such as early detection, risk stratification, and therapeutic targeting, identifying species with direct mechanistic roles is important. Our supervised methods thus reveal disease-relevant signals obscured by abundance-driven variation and confounding in traditional unsupervised approaches, thereby better informing clinical insights.

**Key Points**

- Supervised and covariate-adjusted principal coordinates analysis algorithm (scaPCoA) is a supervised and covariate-adjusted dimension reduction method for microbiome profiling data that simultaneously maximizes association with a clinical outcome while adjusting for confounding variables.
- scaPCoA utilizes pairwise distance to provide an ecological interpretation in terms of beta diversity and is computationally fast.
- scaPCoA addresses key limitations of unsupervised and supervised dimension reduction methods, which can be dominated by abundant microbes associated with confounders, rather than clinically relevant microbial signals.
- scaPCoA utilizes a variable importance metric to identify biologically meaningful microbial features associated with disease outcomes, demonstrated through application to several real microbiome profiling studies.

## Supplementary Material

scaPCoA-supp_bbag590

## Data Availability

Preprocessed data are publicly available through the curated metagenomic data repository and its corresponding software package. An R package, scaPCoA, for our proposed method is available on GitHub at https://github.com/rdeek-lab/scaPCoA.
