# Supplementary material for: Supervised and covariate-adjusted dimension reduction for prediction and visualization using microbiome data
Source: Brief Bioinform. 2026 Oct 27;27(5):bbag590. doi: 10.1093/bib/bbag590 (PMC13712312; doi:10.1093/bib/bbag590)
Supplement: Supplementary file 1 [file scapcoa-supp_bbag590.pdf]

Briefings In Bioinformatics, , pp. 1–15
Paper

Supplementary Material for "Supervised and covariate adjusted dimension reduction for prediction and visualization using microbiome data"

Crystal Zang,[1] Lu Tang[1] and Rebecca A. Deek[1,*]

[1]Department of Biostatistics and Health Data Science, University of Pittsburgh, 130 De Soto St, 15261, PA, USA

*Corresponding author. rdeek@pitt.edu

Abstract

These supplementary materials provide additional information on simulations and real data applications involving scaPCoA and scaPCoA-R.

S1. Null simulation setting

We conducted a null simulation study to evaluate whether the proposed scaPCoA methods introduce spurious outcome-related separation when no true association exists between the microbial features and the outcome. We generated $n = 100$ samples and $p = 200$ microbial features using the same zero-inflated count-generation mechanism as in the main simulation study. The covariates included two continuous variables with weak correlation $\rho = 0.01$ and one categorical covariate with four levels. Under this null setting, the outcome was generated independently of both the microbial features and the covariates. For the binary outcome, we generated $Y_i \sim \text{Bernoulli}(0.5)$. For the continuous outcome, we generated $Y_i \sim N(0, 1)$. Thus, by construction, there was no true predictive signal in the microbial feature matrix for either outcome type.

We evaluated the same set of methods as in the main simulation study, including PCoA, acPCoA, scaPCoA, scaPCoA-R, LASSO, Random Forest, and XGBoost. For PCoA-based methods, prediction models were fit using the estimated principal coordinates as predictors, with and without covariate adjustment. LASSO, Random Forest, and XGBoost were fit using the full clr-transformed microbial feature matrix. As expected under the null setting, none of the methods showed strong predictive performance. For binary outcomes, the AUC values were close to chance level for all methods, with mean AUCs ranging from approximately 0.51 to 0.59, shown in Table S1. For continuous outcomes, the RMSE values were similar across methods. These results suggest that scaPCoA and scaPCoA-R do not produce generalizable artificial outcome-related structure in the held-out data.

Figure S1 further supports this conclusion. In the PC biplots, no clear outcome separation was observed for either binary or continuous outcomes. Consistent with this visual pattern, the silhouette scores for the binary outcome were close to zero for all methods (0.015 for PCoA, 0.022 for acPCoA, 0.086 for scaPCoA, and 0.074 for scaPCoA-R). Notably, the supervised-method scores decreased substantially from 0.339 and 0.403, respectively, in the non-null simulation in Section 3.2 (Figure 2A), indicating that the stronger outcome-group separation observed there depended on the presence of a true outcome-related microbial signal. The partial-correlation summaries revealed a clear distinction between training and test data under the null setting. In the training data, the supervised methods produced moderate correlations between outcome and PC1, reflecting the fact that principal coordinates are estimated using outcome information and may capture random sample-specific

© The Author(s) . Copyright and licence statements to be updated by the publisher during production.

noise when no true signal is present. However, these apparent associations were substantially attenuated in the test data, indicating that the training associations did not generalize to independent samples. Thus, the null simulation demonstrates that supervised ordination does not produce reproducible outcome-related structure in held-out data. These results suggest that the proposed scaPCoA methods do not induce generalizable artificial outcome separation when the microbial features contain no true outcome signal, although training set associations can occur under outcome supervision due to overfitting.

Table S1 Null simulation results comparing model performance in classification and regression tasks with $p = 200$ microbial features and $n = 100$ samples. Binary outcomes are evaluated using area under the curve (AUC; mean(SD)), and continuous outcomes are evaluated using root mean square error (RMSE; mean(SD)). For PCoA, acPCoA, scaPCoA, and scaPCoA-R, generalized linear models for binary outcomes and linear models for continuous outcomes were fit using estimated principal coordinates as predictors, with and without covariates. LASSO, Random Forest, and XGBoost models used all microbial features after clr transformation for prediction.

Outcome	Method	Without Z	With Z
Binary (AUC)	PCoA	0.584 (0.079)	0.588 (0.082)
	acPCoA	0.577 (0.085)	0.594 (0.073)
	scaPCoA	0.593 (0.074)	0.588 (0.077)
	scaPCoA-R	0.580 (0.080)	0.583 (0.072)
	LASSO	0.540 (0.074)	0.538 (0.072)
	Random Forest	0.507 (0.114)	0.507 (0.114)
	XGBoost	0.509 (0.112)	0.516 (0.110)
Continuous (RMSE)	PCoA	1.034 (0.151)	1.061 (0.161)
	acPCoA	1.035 (0.146)	1.055 (0.153)
	scaPCoA	1.095 (0.170)	1.140 (0.181)
	scaPCoA-R	1.095 (0.167)	1.140 (0.178)
	LASSO	1.034 (0.158)	1.028 (0.149)
	Random Forest	1.067 (0.157)	1.065 (0.157)
	XGBoost	1.021 (0.180)	1.018 (0.168)

S2. Evaluation of out-of-sample embedding accuracy

We conducted an additional simulation study to evaluate the accuracy of the Trosset out-of-sample embedding described in Section 2.7 (1). The primary objective was to quantify the additional reconstruction error introduced by embedding new observations while preserving the PCoA configuration estimated from the training data. We compared the out-of-sample procedure with a hypothetical full re-embedding in which PCoA was recomputed after combining the training and test observations.

S2.1. Simulation design

Microbial count data were generated using the same zinLDA-based data-generating mechanism as in the main simulation study, with 200 microbial taxa specified before the removal of all-zero and zero-variance features. We considered training sample sizes $n_{\text{train}} \in \{30, 70, 170\}$ and fixed the test sample size at $n_{\text{test}} = 30$. For each training sample size, we generated 100 independent simulation replicates and retained $m = 3$ principal coordinates. Fixing n_{test} allowed changes in embedding accuracy to be interpreted primarily as a function of the amount of data available for estimating the training PCoA configuration.

Within each replicate, $n_{\text{train}} + n_{\text{test}}$ observations were generated jointly from the baseline zinLDA distribution and randomly divided into the training matrix $\boldsymbol{X}_{\text{train}} \in \mathbb{R}_{+}^{n_{\text{train}} \times p}$ and the matched-test matrix $\boldsymbol{X}_{\text{test}}^{\text{matched}} \in \mathbb{R}_{+}^{n_{\text{test}} \times p}$. An additional shifted-test matrix, $\boldsymbol{X}_{\text{test}}^{\text{shifted}} \in \mathbb{R}_{+}^{n_{\text{test}} \times p}$, was generated independently from a modified zinLDA distribution, as described below.

Given that independently generated zinLDA datasets could retain different subsets of nonzero and nonconstant taxa, the training feature set was used as the reference feature space. Taxa present in the training data but absent

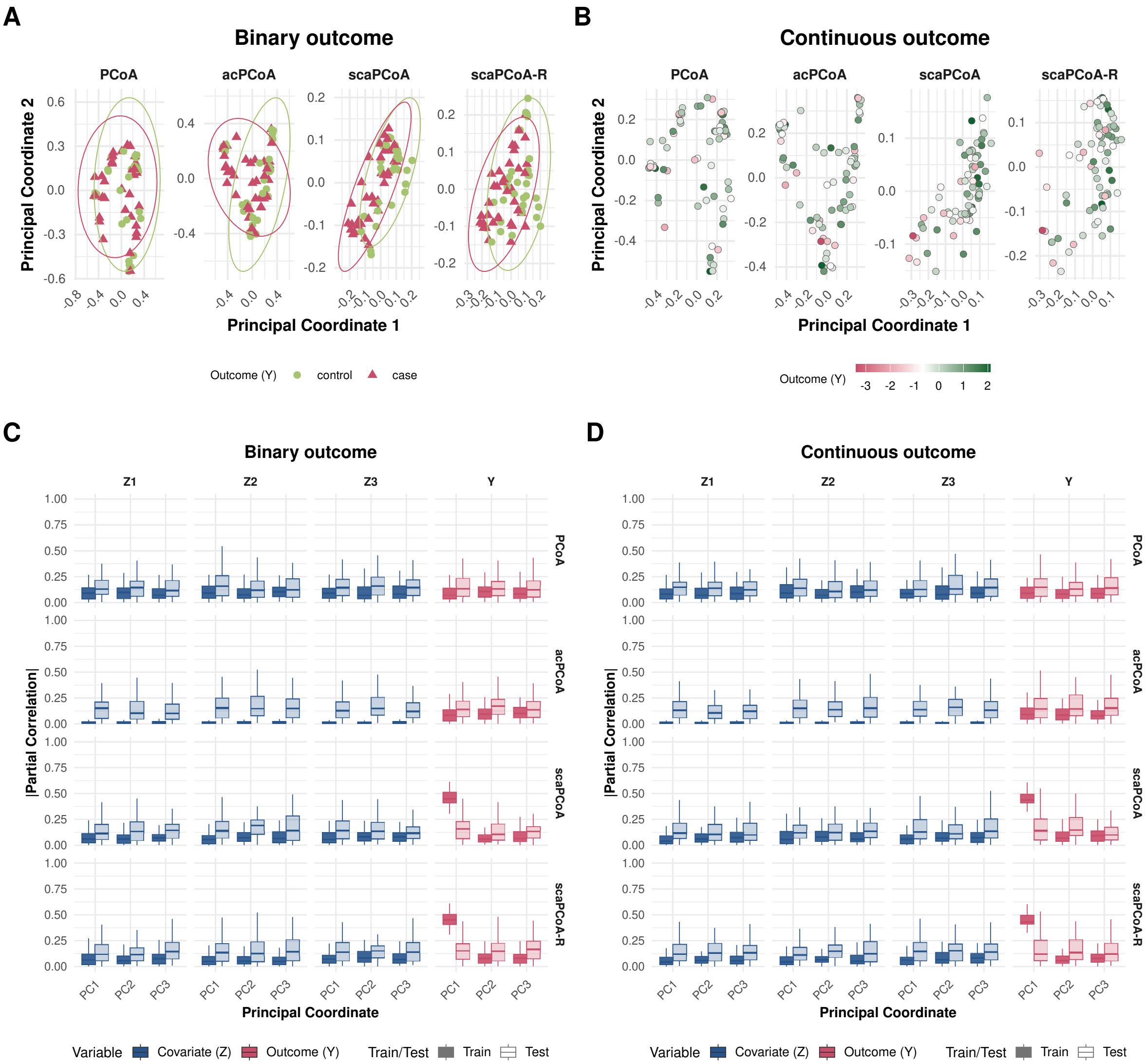

Figure S1 Simulation results for PCoA, acPCoA, scaPCoA, and scaPCoA-R for null setting. **(A)** Principal coordinate (PC) biplots for a binary outcome. Axes show the first two PCs. **(B)** PC biplots, as described in (A), for a continuous outcome. **(C)** Boxplots of the absolute partial correlation between the first three estimated PCs and $\boldsymbol{y}$ (adjusting for $\boldsymbol{Z}$), and between PCs and each $\boldsymbol{Z}$ (adjusting for $\boldsymbol{y}$ and the remaining $\boldsymbol{Z}$), for binary outcome. **(D)** Partial correlation boxplots, as described in (C), for a continuous outcome.

from the shifted test data were assigned counts of zero, whereas taxa present only in the shifted test data were excluded. This ensured that the training and shifted-test matrices contained the same microbial features in the same order.

For each test scenario, Bray-Curtis dissimilarities were computed after combining the training observations with the corresponding test observations. The resulting dissimilarity matrix was partitioned into the training dissimilarity matrix $\boldsymbol{D}_{\text{train}}$, the test dissimilarity matrix $\boldsymbol{D}_{\text{test}}$, and the train-test dissimilarity matrix $\boldsymbol{D}_{\text{train,test}} \in \mathbb{R}^{n_{\text{train}} \times n_{\text{test}}}$. The training PCoA configuration was estimated using only $\boldsymbol{D}_{\text{train}}$, and each test observation was

embedded using the nonlinear Trosset procedure described in Section 2.7. The nonlinear objective was minimized using BFGS (2) with six starting values, and the solution with the smallest objective value was retained.

S2.2. Full re-embedding benchmark

For comparison, we constructed a hypothetical full re-embedding by applying PCoA to the complete dissimilarity matrix obtained after combining the training and test observations. Let $\hat{\boldsymbol{X}}^{\text{Full}}_{\text{train}} \in \mathbb{R}^{n_{\text{train}} \times m}$ and $\hat{\boldsymbol{X}}^{\text{Full}}_{\text{test}} \in \mathbb{R}^{n_{\text{test}} \times m}$ denote the training and test coordinates obtained from this joint PCoA. Unlike the out-of-sample procedure, full re-embedding allows the test observations to affect the centering operation, eigenvalues, eigenvectors, and estimated positions of the training observations. It therefore cannot be used prospectively and was included only as a reference for evaluating the loss in reconstruction accuracy caused by preserving the original training configuration.

S2.3. Train-test distance reconstruction error

For an embedding method $r \in \{\text{OOS}, \text{Full}\}$, let d_{ij} denote the observed Bray-Curtis dissimilarity between training observation i and test observation j, and let $\widehat{d}^{r}_{ij}$ denote their reconstructed Euclidean distance in the m-dimensional coordinate space. For the out-of-sample procedure, $\widehat{d}^{\text{OOS}}_{ij} = \|\hat{\boldsymbol{x}}_i - \hat{\boldsymbol{u}}_j\|_2$, where $\hat{\boldsymbol{x}}_i$ is the training only PCoA coordinate of observation i and $\hat{\boldsymbol{u}}_j$ is the Trosset out-of-sample coordinate of test observation j. For full re-embedding, $\widehat{d}^{\text{Full}}_{ij} = \|\hat{\boldsymbol{x}}^{\text{Full}}_i - \hat{\boldsymbol{x}}^{\text{Full}}_j\|_2$, where both coordinates are obtained from the joint PCoA. For each embedding approach r, we calculate the normalized root mean squared error

$$E_r = \frac{\left\{ \frac{1}{n_{\text{train}} n_{\text{test}}} \sum_{i=1}^{n_{\text{train}}} \sum_{j=1}^{n_{\text{test}}} \left(d_{ij} - \widehat{d}^{r}_{ij} \right)^2 \right\}^{1/2}}{\left\{ \frac{1}{n_{\text{train}} n_{\text{test}}} \sum_{i=1}^{n_{\text{train}}} \sum_{j=1}^{n_{\text{test}}} d^2_{ij} \right\}^{1/2}}. \tag{1}$$

The denominator scales the root mean squared reconstruction error by the root mean square of the observed train-test dissimilarities, allowing results to be compared across simulation settings. We report E_{OOS} and E_{Full} for the Trosset out-of-sample embedding and full re-embedding, respectively.

The primary reconstruction metric was the excess out-of-sample reconstruction error, $\Delta_{\text{OOS}} = E_{\text{OOS}} - E_{\text{Full}}$. Values of Δ_{OOS} near zero indicate that preserving the training configuration introduces little additional train-test reconstruction error relative to joint re-embedding. Positive values of Δ_{OOS} indicate greater error under out-of-sample embedding. Negative values are also possible because full PCoA does not directly minimize the train-test component of the reconstruction error in isolation.

S2.4. Matched and shifted test distributions

We evaluated embedding accuracy under matched and shifted test distributions. In the matched setting, the training and test observations were generated jointly from the same baseline zinLDA distribution using $K = 5$, $\alpha = 0.1$, $\pi = 0.7$, $a = 0.6$, and $b = 10$, and were subsequently divided into their respective datasets.

In the shifted setting, the test observations were generated independently using a second zinLDA simulation. At the hyperparameter level, the number of latent subcommunities, zero-inflation parameter, and generalized Dirichlet parameters were held fixed, while the symmetric Dirichlet concentration parameter governing the sample-specific subcommunity proportions was increased from $\alpha = 0.1$ to $\alpha_{\text{shift}} = 0.5$. Thus, the shifted distribution used $K = 5$, $\alpha_{\text{shift}} = 0.5$, $\pi = 0.7$, $a = 0.6$, and $b = 10$.

Increasing α produces less concentrated and more evenly mixed sample-specific subcommunity proportions. Because the shifted test set was generated independently under the modified zinLDA hyperparameter configuration, its subcommunity proportions, taxon probabilities, structural-zero indicators, and covariate realizations were all newly sampled. The matched and shifted scenarios shared the same training dataset within each simulation replicate but used separate test datasets. They therefore permitted comparison of out-of-sample embedding under matched and shifted data-generating distributions, but did not constitute an observation-level paired comparison of the same held-out test samples.

S2.5. Results

Figure S2 presents the excess train-test distance reconstruction error, $\Delta_{\text{OOS}} = E_{\text{OOS}} - E_{\text{Full}}$, across training sample sizes under the matched and shifted test distributions. Under the matched setting, the excess reconstruction error was centered near zero for all training sample sizes, indicating that the Trosset out-of-sample procedure achieved reconstruction accuracy comparable to full re-embedding when the training and test data arose from the same distribution.

Under the shifted setting, the excess reconstruction error was consistently positive, reflecting the additional difficulty of embedding observations generated from a different microbial distribution while preserving the original training configuration. However, the magnitude and variability of the excess error decreased as the training sample size increased. This trend is expected because a larger training sample provides a more stable estimate of the underlying PCoA configuration, thereby improving the robustness of out-of-sample embedding under moderate distributional shift.

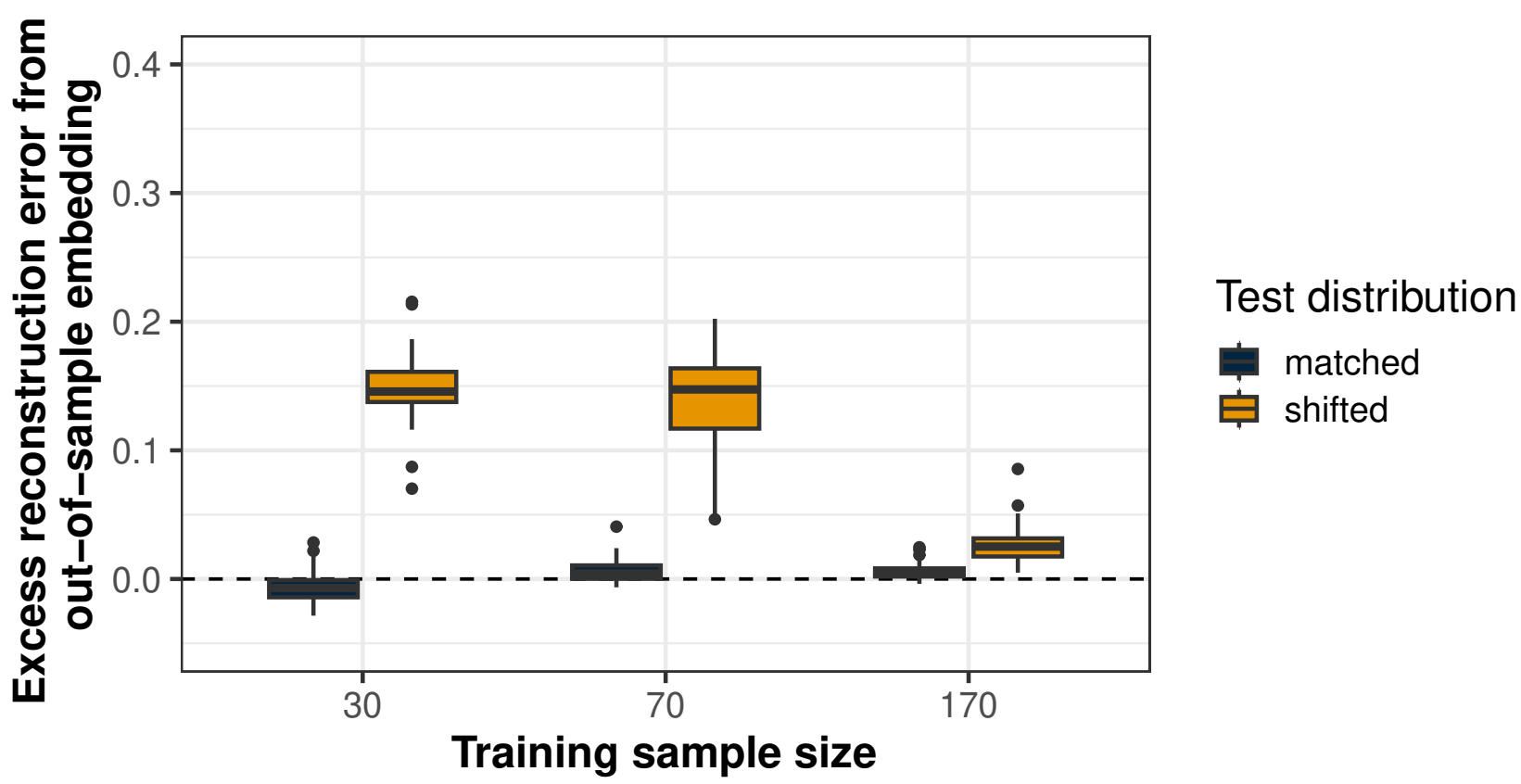

Figure S2 Excess train-test reconstruction error for the Trosset out-of-sample embedding relative to full re-embedding across training sample sizes, based on 100 simulation replicates with a fixed test sample size of 30. Matched test samples were generated jointly with the training samples from the baseline zinLDA distribution using $\alpha = 0.1$. Shifted test samples were generated independently from a modified zinLDA distribution using $\alpha = 0.5$, while the remaining zinLDA hyperparameters and sequencing-depth distribution were held fixed. Values near zero indicate similar train-test reconstruction accuracy between out-of-sample embedding and full re-embedding.

S3. Comparison to existing methods in simulation

S3.1. PCA-based methods

We further compared our proposed supervised methods, scaPCoA and scaPCoA-R with two PCA-based approaches that operate under Euclidean distance assumptions: PCA and PeDecURe (3). PCA is an unsupervised method without nuisance variation adjustment, while PeDecURe is a supervised method with nuisance variation adjustment. Both methods were applied directly to microbial counts without further normalization.

As shown in Figure S3A/B, both PCA and PeDecURe failed to capture outcome-relevant variation, as indicated by the substantial overlap between subjects from the binary outcome groups and the absence of a clear gradient in continuous outcomes in the lower-dimensional representation. Figure S3C/D further demonstrates that PCA did not identify principal components associated with the outcome in either training or out-of-sample datasets. Although PeDecURe extracted outcome-associated components within the training data, its performance deteriorated in the out-of-sample data, exhibiting a marked reduction in absolute partial correlation.

For out-of-sample outcome prediction, using the estimated principal components, both PCA and PeDecURe underperformed compared with our proposed scaPCoA methods. While PeDecURe appeared to estimate principal components that are highly correlated with the outcome in training data (median absolute correlation of

0.61; Figure S3C/D), this correlation dropped substantially to 0.18 in the testing data, leading to a decline in predictive performance. For the binary outcome, PCA achieved mean AUCs of 0.725 (SD=0.114) without covariate adjustment and 0.741 (SD=0.100) with covariates, whereas PeDecURe achieved mean AUCs of 0.628 (SD=0.107) without covariates and 0.619 (SD=0.098) with covariates. For the continuous outcome, PCA yielded a mean RMSE of 1.038 (SD=0.229) without covariates and 1.005 (SD=0.218) with covariates, while PeDecURe resulted in a mean RMSE of 3.496 (SD=2.979) and 1.412 (SD=0.315) without and with covariates, respectively.

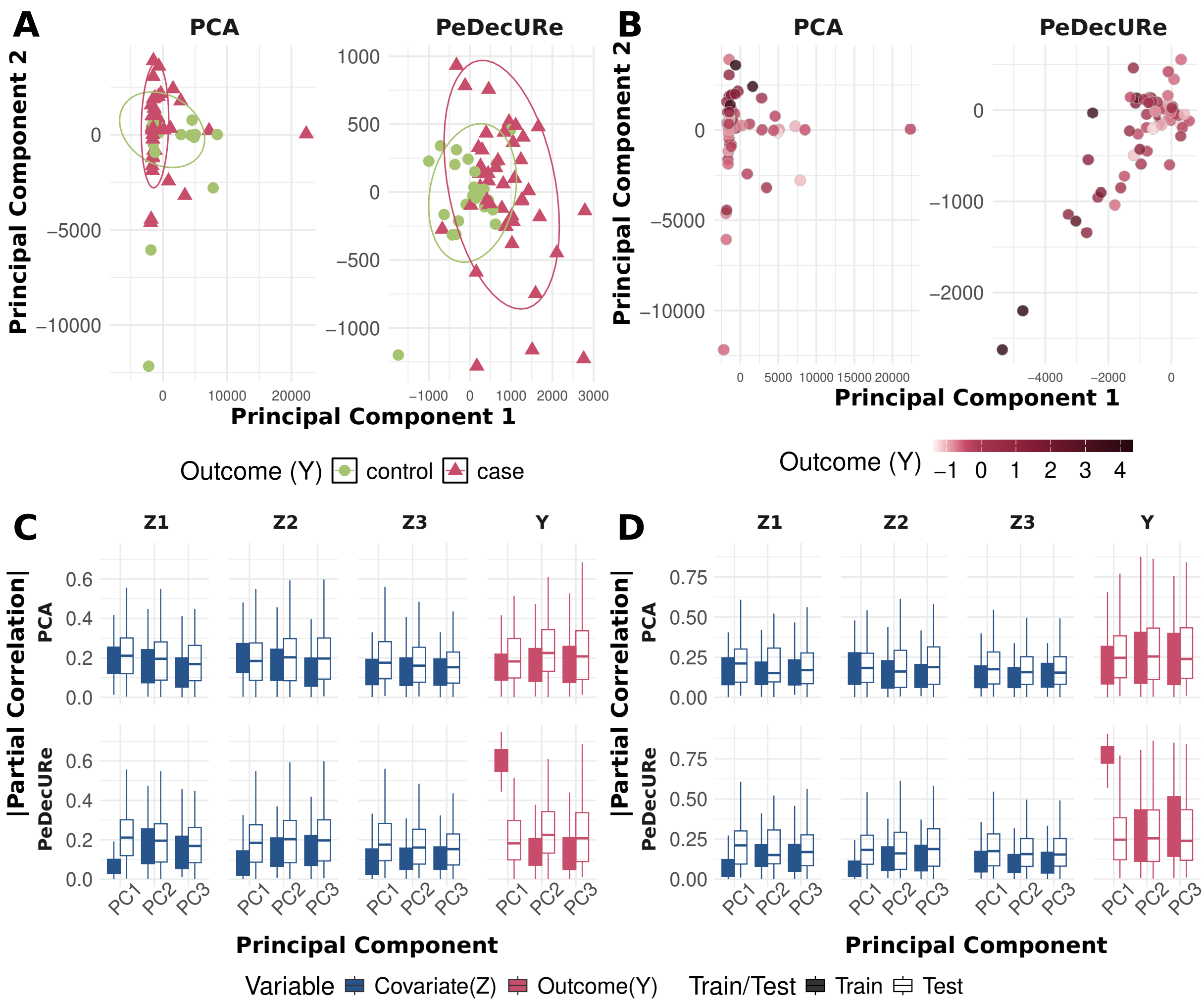

Figure S3 Simulation results for PCA and PeDecURe. **(A)** Principal component biplots for a binary outcome. Axes show the first two principal components. (**B**) Principal component biplots, as described in (A), for a continuous outcome. **(C)** Boxplots of the absolute partial correlation between the first three estimated principal components and $\boldsymbol{y}$ (adjusting for $\boldsymbol{Z}$), and between principal components and each $\boldsymbol{Z}$ (adjusting for $\boldsymbol{y}$ and the remaining $\boldsymbol{Z}$), for binary outcome. **(D)** Partial correlation boxplots, as described in (C), for a continuous outcome.

S3.2. Nonlinear methods

For visualization, we evaluated additional nonlinear methods t-distributed stochastic neighbor embedding (t-SNE) (4) and Uniform Manifold Approximation and Projection (UMAP) (5). As expected from unsupervised approaches, visualizations from both methods applied to our simulation scenarios (Figure S4) showed no clear

separation between outcome groups, as these methods prioritize preserving data structure rather than outcome-related variation. These results mirrored those from unsupervised PCA, PCoA and acPCoA (Figures 2A/B and S3A/B).

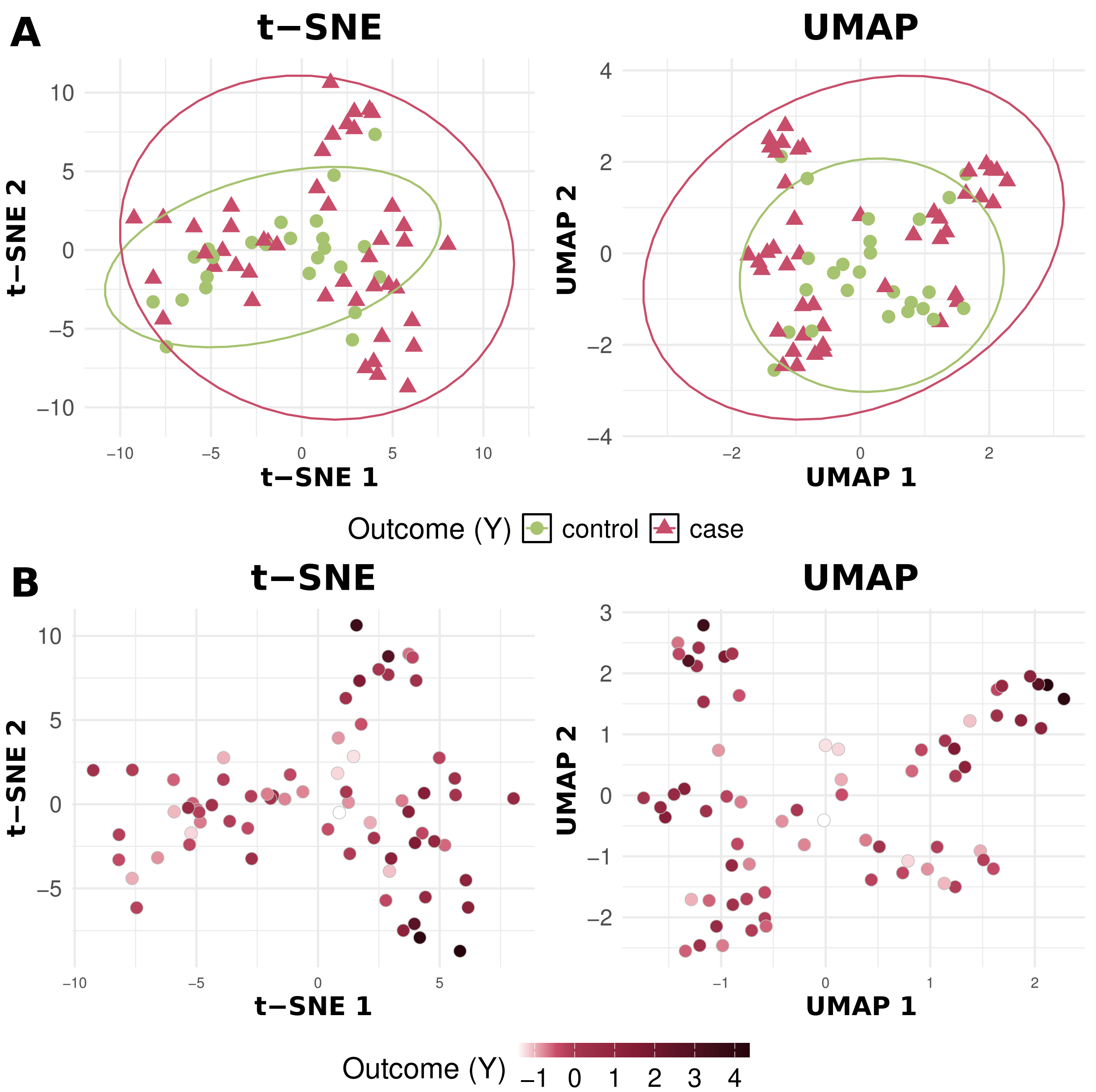

Figure S4 Biplots of simulated data using t-SNE and UMAP. **(A)** Biplots for a binary outcome. **(B)** Biplots for a continuous outcome.

S4. Comparison to existing methods using several colorectal cancer metagenomic profiling studies

S4.1. PCA-based methods

In our real data application, involving five metagenomic sequencing datasets, visualization of the first two principal components (Figure S5A) shows substantial overlap between the CRC outcome groups under both PCA and PeDecURe, suggesting that the Euclidean distance assumption produced projections that fail to separate outcome-relevant variation.

For out-of-sample disease status prediction using the estimated principal components, both PCA and PeDecURe underperformed compared to the scaPCoA methods. Although the principal components estimated by PeDecURe showed a moderate association with CRC in the training data (median absolute partial correlation of 0.46), this association dropped to 0.08 in testing data (Figure S5B). Across five-fold cross-validation, PCA achieved mean AUCs of 0.575 (SD=0.053) without adjusting for sex, age, and BMI, and 0.594 (SD=0.055) with covariate adjustment. PeDecURe achieved mean AUCs of 0.551 (SD=0.050) without covariates and 0.562 (SD=0.066) with covariates.

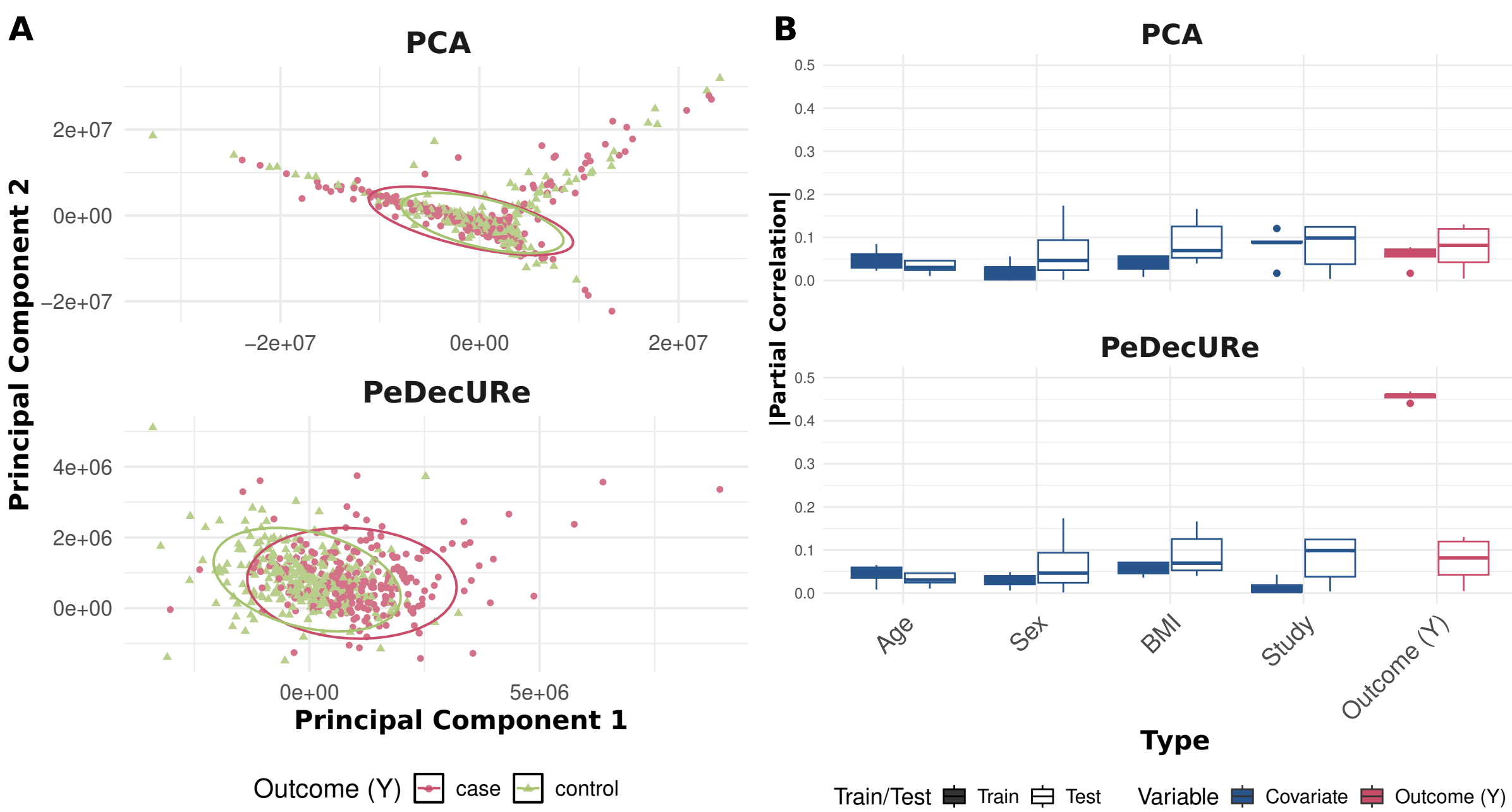

Figure S5 (**A**) Principal component biplots of metagenomic data from case-control colorectal cancer studies using PCA and PeDecURe. (**B**) Boxplots of the absolute partial correlations between the first principal component and covariates or binary disease status in training and testing data across five-fold cross-validation.

S4.2. Nonlinear methods

Consistent with our simulation findings, we observed no separation between subjects with CRC and healthy controls (Figure S6) under both t-SNE and UMAP projections. These results mirrored those from PCA, PCoA and acPCoA (Figures 3A and S5A), reinforcing that unsupervised methods, regardless of their sophistication in capturing nonlinear relationships, consistently fail to reveal outcome-relevant patterns, attributable to the lack of explicitly incorporating outcome information into their dimension reduction.

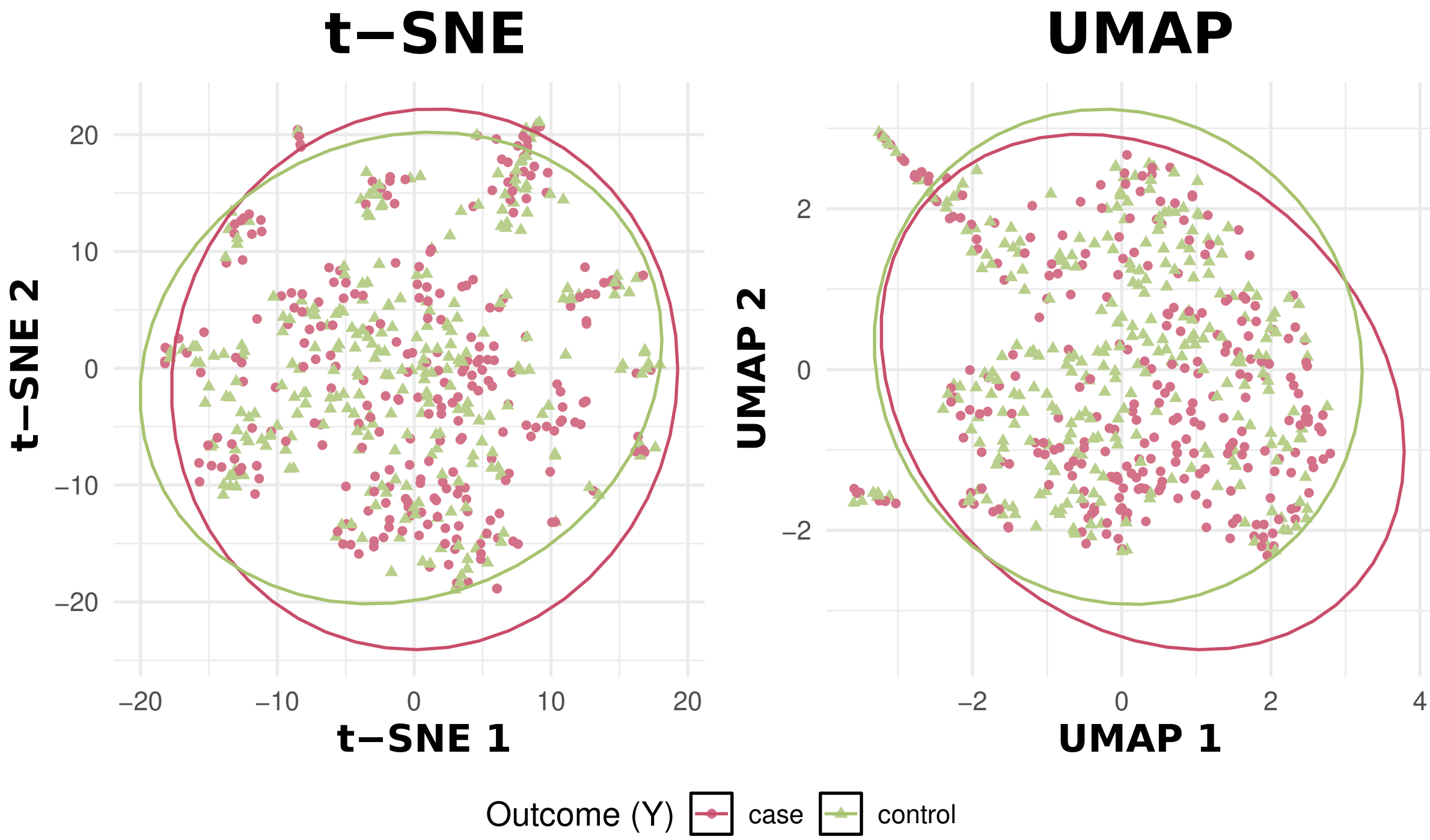

Figure S6 Biplots of metagenomic data from case-control colorectal cancer studies using t-SNE and UMAP.

S5. Variance explained by principal coordinates

To assess the number of supervised coordinates needed for downstream analysis, we examined the scaPCoA eigenvalue distribution across 100 simulated binary outcome datasets with $n = 100$ samples and $p = 200$ microbial features. Within each simulation replicate, the coordinates were ordered by their corresponding eigenvalues, and the proportion of fitted coordinate variability explained by each coordinate was calculated. As shown in Figure S7, the first coordinate accounted for nearly all of the fitted coordinate variability, while the contributions of the remaining coordinates were substantially smaller. Thus, retaining the first three coordinates captures the dominant supervised structure while providing a parsimonious representation for visualization and prediction.

S6. Quantifying the contribution of microbial features to low-dimensional embeddings

Understanding which microbial features drive the predictive performance of proposed methods, scaPCoA and scaPCoA-R, provides additional biological insights and interpretability. However, deriving feature importance for PCoA-based methods is not straightforward. Thus, to quantify the contribution of each microbial feature to the PCs, we construct a microbial feature importance score using microbiome features $\boldsymbol{X} \in \mathbb{R}^{n \times p}$, PCs $\check{\boldsymbol{X}} \in \mathbb{R}^{n \times m}$, and eigenvalues $\boldsymbol{\theta} \in \mathbb{R}^{m}$.

We compute the pairwise Spearman rank correlations between the microbial features and PCs: $\boldsymbol{S} = \mathrm{Corr}_{sp}(\boldsymbol{X}, \check{\boldsymbol{X}})$, where s_{kt} represents the rank correlation between the kth feature and t^{th} PC. Legendre and Legendre (6) introduced a scaled loading matrix that emphasizes the contributions along higher-variance PCs. Accordingly, we define: $\tilde{\boldsymbol{S}} = \boldsymbol{S} \cdot \mathrm{diag}\left(\sqrt{\boldsymbol{\theta}}\right)$, where $\tilde{s}_{kt} = s_{kt}\sqrt{\theta_t}$. To quantify the overall contribution of feature $\boldsymbol{x}_k$ across all PCs, we define its importance as $\boldsymbol{s}_k^{\mathrm{sum}} = \sum_{t=1}^{m} |\tilde{s}_{kt}|$.

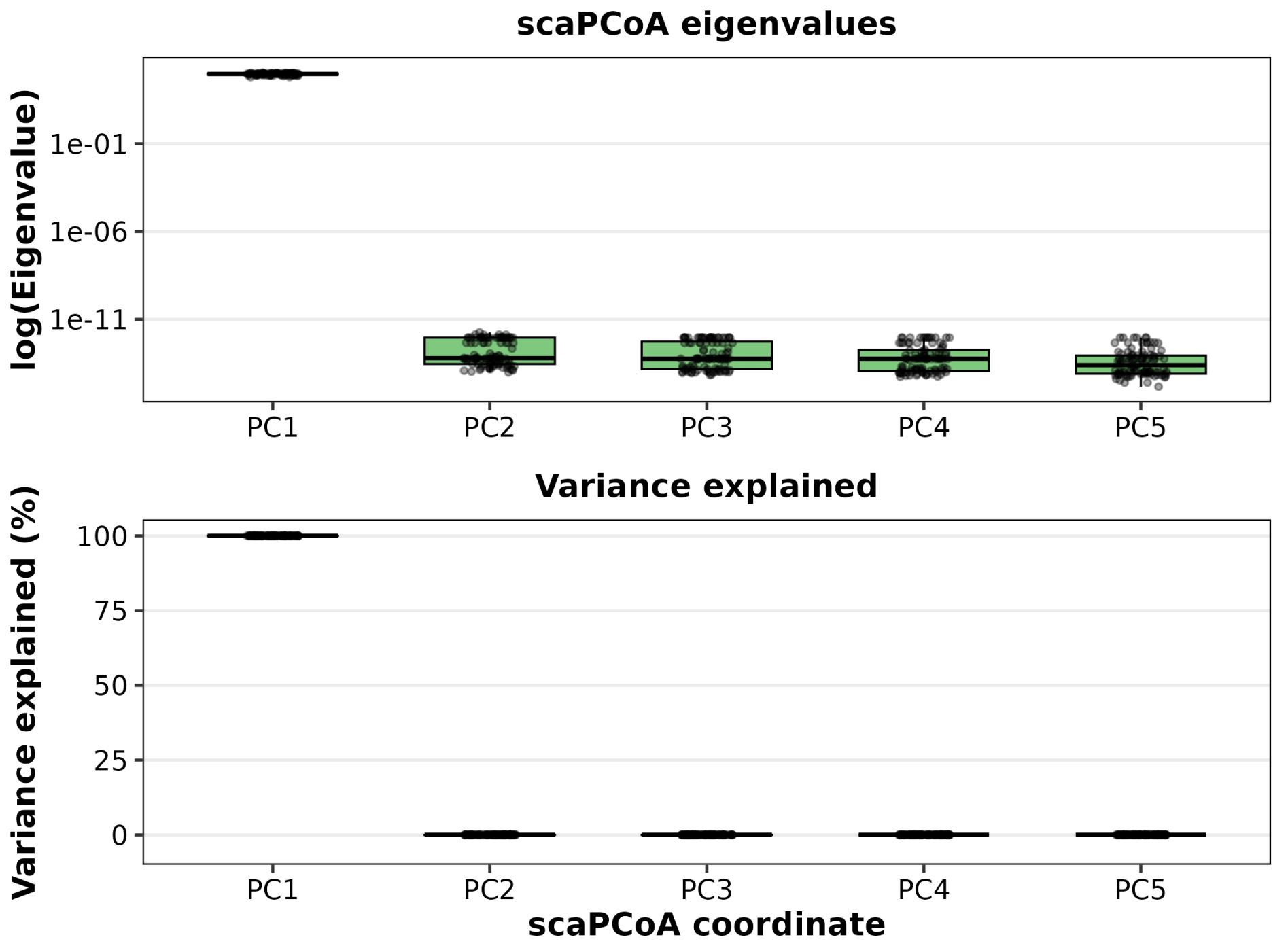

Figure S7 Distribution of scaPCoA eigenvalues and variance explained across 100 simulated datasets with $n = 100$ and $p = 200$. Eigenvalues are shown on a log-scale. The first scaPCoA coordinate explains nearly all of the fitted coordinate variability, supporting the use of a small number of supervised coordinates in downstream prediction.

S7. Application to schizophrenia microbiome profiling study

S7.1. Study data and preprocessing

We applied scaPCoA to microbiome profiling data from a case-control study investigating the gut microbiome composition in schizophrenia patients (7). The study enrolled 97 adults from Brisbane, Australia, 72 of whom had a schizophrenia diagnosis and 25 healthy controls. Stool samples underwent shotgun metagenomic sequencing on an Illumina NovaSeq6000 platform with 300-bp paired-end reads. Microbial composition was profiled using the Microba Community Profiler with direct taxonomic binning, yielding 1431 species-level features. We computed pairwise Bray-Curtis dissimilarity to quantify the β-diversity. Covariates for adjustment in scaPCoA included age, sex, BMI, stool type, physical activity, and diet. Stool type was assessed using the Bristol Stool Scale. Physical activity was measured using the International Physical Activity Questionnaire (IPAQ) short form and calculated as total metabolic equivalent (MET)-minutes per week (8). Dietary patterns were assessed using the Australian Eating Survey (9) and summarized following procedures described in Vasileva et al.(7), with the top 4 principal components explaining 49% of dietary variance included as covariates.

To address class imbalance, we applied the Synthetic Minority Over-sampling Technique (SMOTE) (10) to augment the control group. SMOTE created synthetic samples through feature-space interpolation. For each minority class sample, it identified 5 nearest neighbors and generated new samples by randomly interpolating along the line segments connecting the sample to its neighbors. This approach produced novel, but realistic microbiome profiles that preserved the underlying distributional characteristics of the control group while avoiding duplication. The procedure generated 50 synthetic control samples, resulting in a balanced dataset of 75 controls and 72 schizophrenia cases for analysis.

We compared our proposed methods scaPCoA(-R) to unsupervised methods PCoA and acPCoA, and machine learning methods LASSO, Random Forest and XGBoost. The model performance and evaluation approaches are the same as those in Section 3.3.

S7.2. Visualizing patterns of the global microbiome

Visualizations of the data using the first two principal coordinates (Figure S8A) demonstrate that the proposed scaPCoA and scaPCoA-R methods achieved clear separation between schizophrenia and healthy control participants. In contrast, the unsupervised methods, PCoA and acPCoA, showed substantial overlap between the two groups. Consistent with these patterns, the mean silhouette widths were 0.465 for scaPCoA and 0.492 for scaPCoA-R, compared with 0.131 for PCoA and 0.052 for acPCoA. In alignment with our findings, MiRKAT identified a statistically significant association between microbiome composition and schizophrenia status after adjusting for covariates ($p < 0.001$), thus supporting the disease-related variation captured by the supervised embeddings (11).

Furthermore, unsupervised PCoA and acPCoA failed to extract PCs that were meaningfully correlated with the outcome (Figure S8B). For PCoA, PC1 was only slightly more correlated with the outcome (median absolute partial correlation of 0.3) than with covariates. Although acPCoA reduced correlations with covariates in training, these correlations inflated in testing, and its association with the outcome remained weak (median absolute partial correlation of 0.16 in testing). In contrast, scaPCoA and scaPCoA-R identified a first principal coordinate that exhibited strong associations with the outcome, achieving median absolute partial correlations of 0.73 and 0.75 in testing, which are substantially higher than those of the unsupervised methods.

S7.3. Prediction of schizophrenia status

For all PC-based methods, we built generalized linear models using the top three estimated principal coordinates as predictors, with and without adjustment for age and sex to predict schizophrenia disease status. Age was standardized to enable direct comparison of model coefficients. Across the five cross-validation folds, scaPCoA and scaPCoA-R achieved higher test AUCs than PCoA and acPCoA (Table S2). When covariates were not included in the prediction model, scaPCoA and scaPCoA-R achieved mean AUCs of 0.929 and 0.926, respectively, which were nearly equivalent to LASSO, Random Forest, and XGBoost, whose mean AUCs ranged from 0.923 to 0.934. Adding age and sex in the prediction model resulted in similar, slightly lower AUCs for scaPCoA and scaPCoA-R, indicating that these variables provided little additional predictive information beyond that already captured by the supervised microbial coordinates in this dataset.

Table S2 Schizophrenia study results comparing out-of-fold predictive performance, reported as the mean AUC (SD) across the held-out test folds from five-fold cross-validation. For PCoA, acPCoA, scaPCoA, and scaPCoA-R, a generalized linear model was used to predict the outcome from the estimated principal coordinates, either without or with covariates age and sex. LASSO, Random Forest, and XGBoost used the microbial features directly as predictors, either without or with covariates.

Method	Without Covariates	With Covariates
PCoA	0.892 (0.103)	0.886 (0.095)
acPCoA	0.573 (0.088)	0.602 (0.118)
scaPCoA	0.929 (0.091)	0.926 (0.082)
scaPCoA-R	0.926 (0.093)	0.921 (0.091)
LASSO	**0.934** (0.047)	**0.934** (0.047)
Random Forest	0.931 (0.054)	0.923 (0.066)
XGBoost	0.923 (0.051)	0.932 (0.056)

Figure S9A illustrates the effect sizes of predictors, captured by the absolute value of the model coefficients, reflecting their association with schizophrenia status across all models. The second PC of unsupervised PCoA had the largest model coefficient and all PCs and covariates had similar coefficients from unsupervised acPCoA, indicating similar contributions for predicting schizophrenia status. In contrast, for supervised methods, PC1

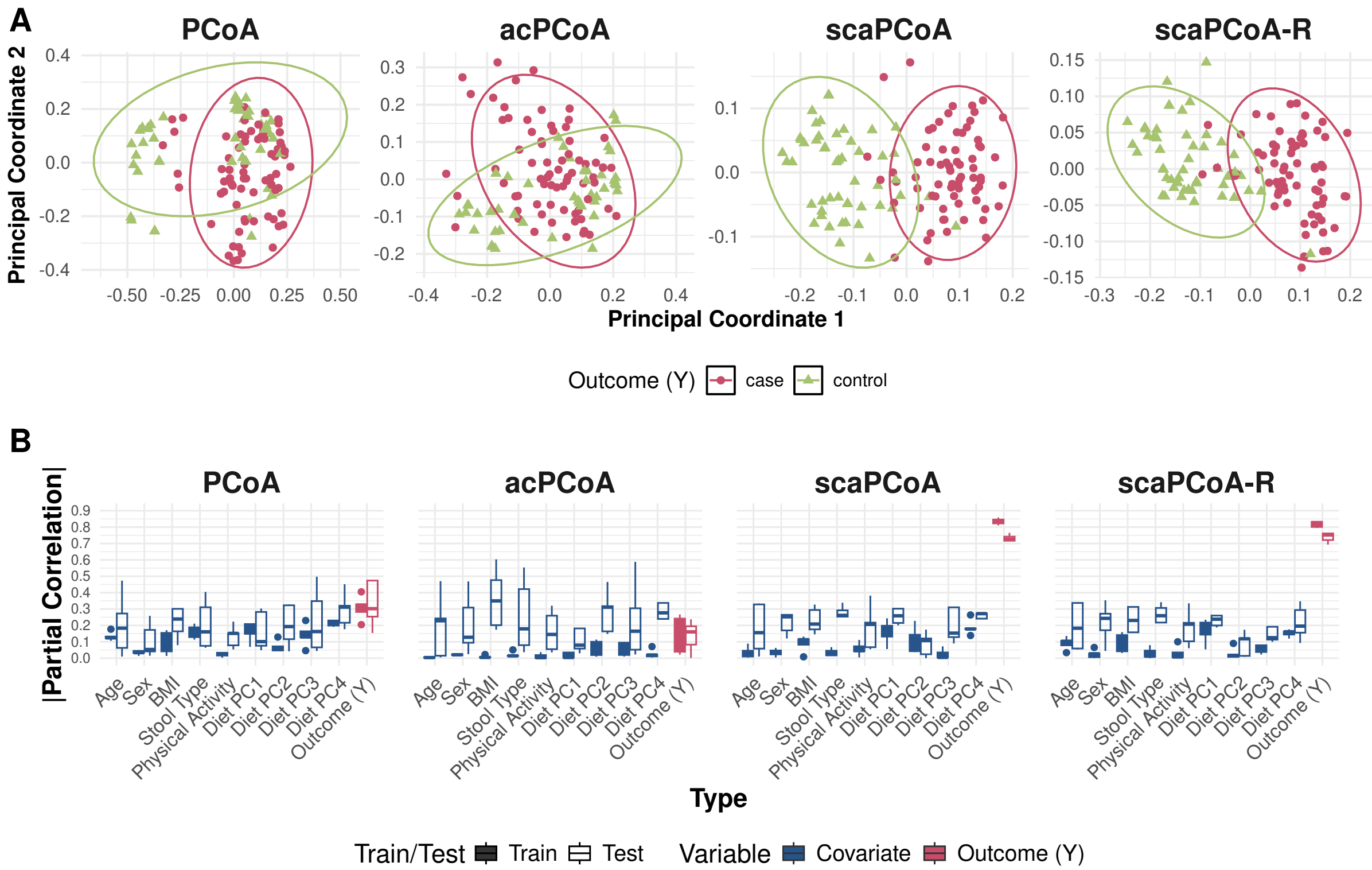

Figure S8 **(A)** Principal coordinate biplots from a case-control schizophrenia study for PCoA, acPCoA, scaPCoA and scaPCoA-R. Points are shaped and colored by binary schizophrenia outcome status. **(B)** Boxplots of the absolute partial correlations between the first principal coordinate and covariates or binary schizophrenia status in training and testing data across five-fold cross-validation.

contributed substantially more to predicting schizophrenia status than the covariates, as indicated by their larger absolute coefficients (Figure S9A).

S7.4. Microbial variable importance scores

We present the fifteen microbial features with the largest median scaled s^{sum} values across the cross-validation folds. As shown in Figure S10, PCoA and acPCoA highlighted common gut taxa, including *Bacteroides vulgatus*, *Bacteroides uniformis*, and *Prevotella copri*, all of which are prevalent members of the human gut microbiome (12). In contrast, scaPCoA and scaPCoA-R highlighted several less-studied microbial taxa, including *Blautia A sp.7810* and multiple unclassified members of the family *Oscillospiraceae*. The label *Blautia A sp.7810* denotes an unnamed species within the provisional Genome Taxonomy Database genus *Blautia A*, whereas the *Oscillospiraceae* features could not be resolved to a named genus. Unsupervised PCoA and acPCoA also identified other species within *Blautia A*, including *Blautia A* sp.000433815, but did not prioritize the same species-level feature identified by the supervised methods.

Previous human studies have repeatedly associated the genus *Blautia* with schizophrenia. Across multiple schizophrenia cohorts, *Blautia*-related taxa have generally been reported at lower abundance than in healthy controls, although the direction of association varies by species, disease stage, and antipsychotic treatment (13; 14; 7). Furthermore, *Blautia* species participate in carbohydrate fermentation and short-chain-fatty-acid production. In an experimental mouse model, *Blautia* was shown to promote colonic mucus growth through the production of acetate and propionate and activation of the short-chain-fatty-acid receptor Ffar2 (15). Although

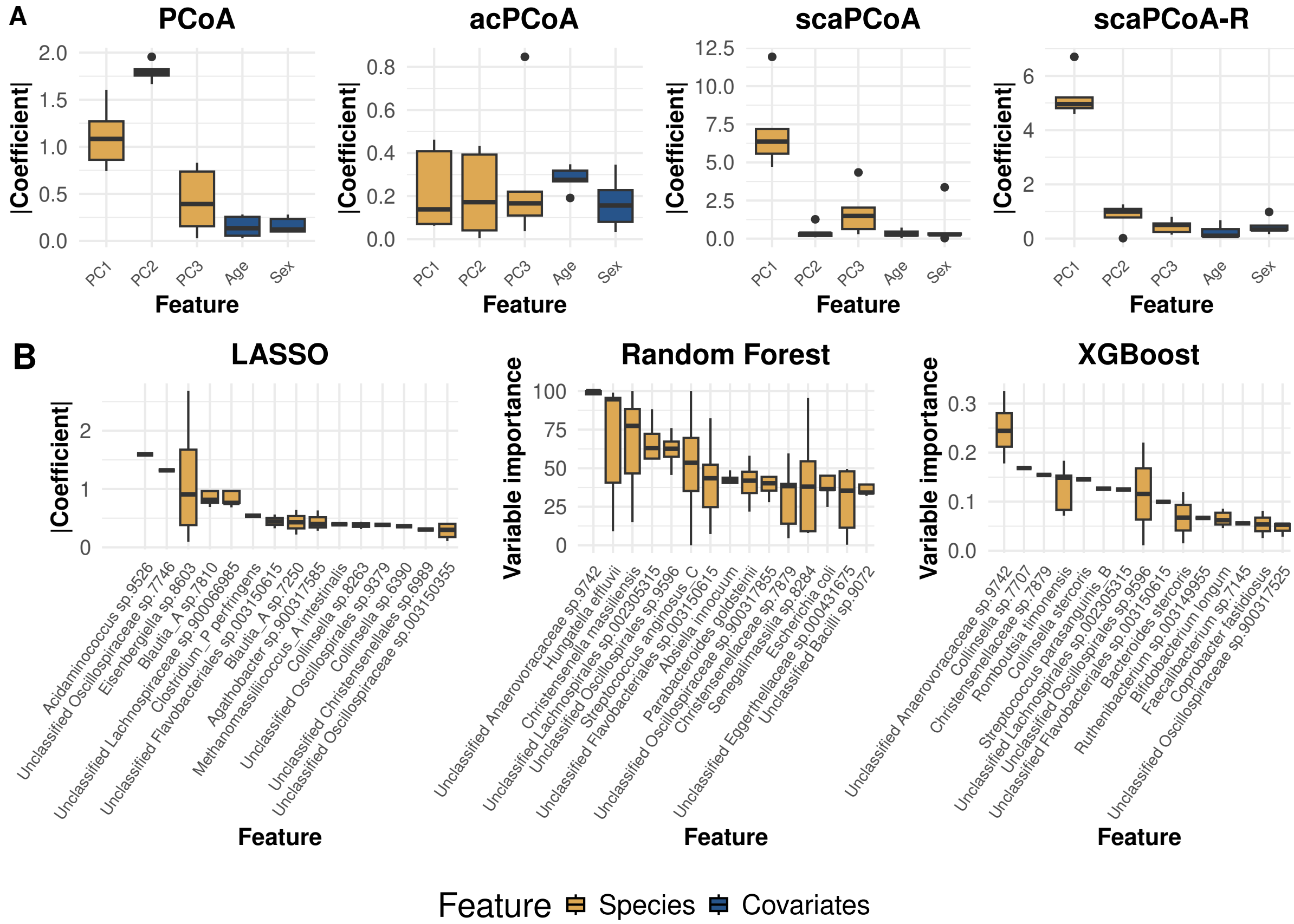

Figure S9 (**A**) Absolute coefficients from generalized linear models for predicting schizophrenia status using the principal coordinates estimated by PCoA, acPCoA, scaPCoA, and scaPCoA-R, together with covariates. **(B)** Feature importance for predicting schizophrenia status using all microbial features and covariates, represented by absolute LASSO coefficients, Random Forest variable importance, and XGBoost gain. The 15 most important microbial features within each method are shown.

this mechanism has not been established in schizophrenia, it provides a biologically plausible pathway through which altered *Blautia* abundance could affect intestinal barrier function and microbiota-gut-brain signaling.

The *Oscillospiraceae* taxa have also been associated with schizophrenia. A previous study reported depletion of *Oscillospira* in patients with schizophrenia compared with healthy controls (16). The identification of multiple unclassified *Oscillospiraceae* species by scaPCoA and scaPCoA-R therefore highlights a comparatively understudied component of the schizophrenia-associated gut microbiome and generates specific candidates for future taxonomic and functional investigation.

At the exact species-feature level, the leading contributors selected by scaPCoA and scaPCoA-R generally differ from the top predictors selected by LASSO, Random Forest, or XGBoost. This difference suggests that supervised ordination and conventional predictive models may prioritize complementary microbial signals: scaPCoA identifies taxa that contribute to community-level outcome-associated variation, whereas the machine-learning methods select individually discriminative predictors.

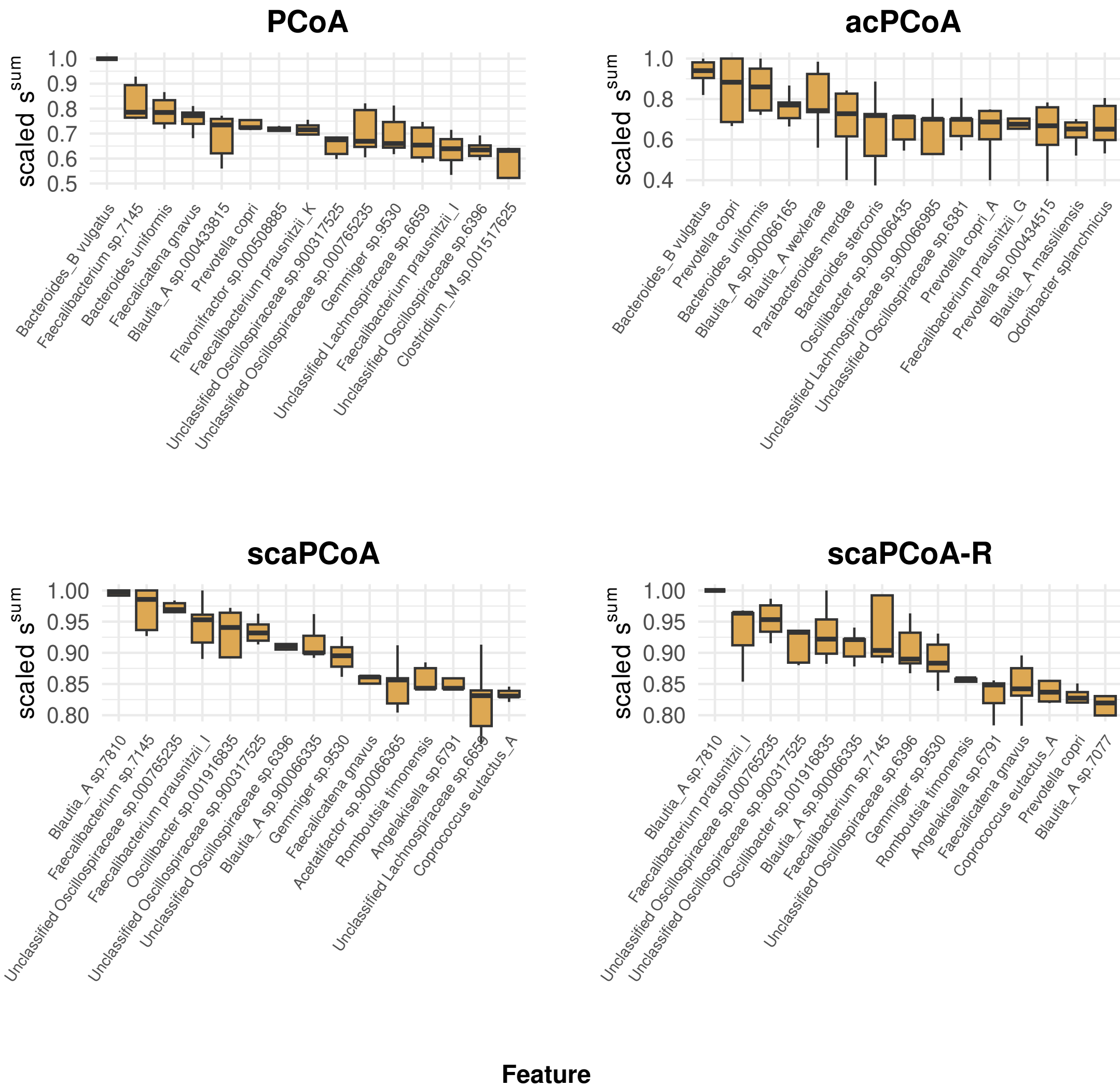

Figure S10 Top microbial features contributing to principal coordinates, and therefore schizophrenia status classification. Microbes with the highest contributions to the estimated principal coordinates (s^{sum}), displayed for PCoA, acPCoA, scaPCoA, and scaPCoA-R. Importance scores are rescaled between 0 and 1 within each method.

REFERENCES

1. Trosset MW, Priebe CE. The out-of-sample problem for classical multidimensional scaling. Computational Statistics & Data Analysis. 2008;52(10):4635-42.
2. Nocedal J, Wright SJ. Numerical Optimization. 2nd ed. New York: Springer; 2006.
3. Weinstein SM, Davatzikos C, Doshi J, Linn KA, Shinohara RT. Penalized decomposition using residuals (PeDecURe) for feature extraction in the presence of nuisance variables. Biostatistics. 2023;24(3):653-68.
4. van der Maaten L, Hinton G. Visualizing Data using t-SNE. Journal of Machine Learning Research. 2008;9(86):2579-605. Available from: `http://jmlr.org/papers/v9/vandermaaten08a.html`.
5. McInnes L, Healy J, Melville J. UMAP: Uniform manifold approximation and projection for dimension reduction. arXiv preprint arXiv:180203426. 2018.
6. Legendre P, Legendre L. Numerical ecology. 2nd ed. No. 20 in Developments in Environmental Modelling. Amsterdam: Elsevier; 1998.
7. Vasileva SS, Yang Y, Baker A, Siskind D, Gratten J, Eyles D. Associations of the Gut Microbiome With Treatment Resistance in Schizophrenia. JAMA Psychiatry. 2024;81(3):292-302.
8. IPAQ Research Committee. Guidelines for data processing and analysis of the International Physical Activity Questionnaire (IPAQ)–short and long forms; 2005.
9. Collins CE, Boggess MM, Watson JF, Guest M, Duncanson K, Pezdirc K, et al. Reproducibility and comparative validity of a food frequency questionnaire for Australian adults. Clinical Nutrition. 2014;33(5):906-14.
10. Chawla NV, Bowyer KW, Hall LO, Kegelmeyer WP. SMOTE: synthetic minority over-sampling technique. Journal of Artificial Intelligence Research. 2002;16:321-57.
11. Zhao N, Chen J, Carroll IM, Ringel-Kulka T, Epstein MP, Zhou H, et al. Testing in microbiome-profiling studies with MiRKAT, the microbiome regression-based kernel association test. American Journal of Human Genetics. 2015;96(5):797-807.
12. Yang J, et al. Species-Level Analysis of Human Gut Microbiota With Metataxonomics. Frontiers in Microbiology. 2020;11:2029.
13. Yuan X, et al. Gut microbial biomarkers for the treatment response in first-episode, drug-naive schizophrenia: a 24-week follow-up study. Translational Psychiatry. 2021;11:422.
14. Zheng P, et al. The gut microbiome from patients with schizophrenia modulates the glutamate-glutamine-GABA cycle and schizophrenia-relevant behaviors in mice. Science Advances. 2019;5(2):eaau8317.
15. Holmberg SM, Feeney RH, Prasoodanan P K V, Puértolas-Balint F, Singh DK, Wongkuna S, et al. The gut commensal Blautia maintains colonic mucus function under low-fiber consumption through secretion of short-chain fatty acids. Nature Communications. 2024;15:3502.
16. Wei N, Ju M, Su X, Zhang Y, Huang Y, Rao X, et al. Transplantation of Gut Microbiota Derived From Patients With Schizophrenia Induces Schizophrenia-Like Behaviors and Dysregulated Brain Transcript Response in Mice. Schizophrenia. 2024;10:44.
